# Synthesis and Modulation of Low-Dimensional Transition Metal Chalcogenide Materials via Atomic Substitution

**DOI:** 10.1007/s40820-024-01378-5

**Published:** 2024-03-28

**Authors:** Xuan Wang, Akang Chen, XinLei Wu, Jiatao Zhang, Jichen Dong, Leining Zhang

**Affiliations:** 1 Key Laboratory of Cluster Science, Ministry of Education of China, Beijing Key Laboratory of Photoelectronic and Electrophonic Conversion Materials, School of Chemistry and Chemical Engineering, Beijing Institute of Technology, Beijing, 100081 People’s Republic of China; 2 Beijing National Laboratory for Molecular Sciences, Key Laboratory of Organic Solids, Institute of Chemistry Chinese Academy of Sciences, Beijing, 100190 People’s Republic of China

**Keywords:** Transition metal chalcogenides, Atomic substitution, Ion exchange, Low-dimensional materials, Controllable synthesis

## Abstract

In recent years, low-dimensional transition metal chalcogenide (TMC) materials have garnered growing research attention due to their superior electronic, optical, and catalytic properties compared to their bulk counterparts. The controllable synthesis and manipulation of these materials are crucial for tailoring their properties and unlocking their full potential in various applications. In this context, the atomic substitution method has emerged as a favorable approach. It involves the replacement of specific atoms within TMC structures with other elements and possesses the capability to regulate the compositions finely, crystal structures, and inherent properties of the resulting materials. In this review, we present a comprehensive overview on various strategies of atomic substitution employed in the synthesis of zero-dimensional, one-dimensional and two-dimensional TMC materials. The effects of substituting elements, substitution ratios, and substitution positions on the structures and morphologies of resulting material are discussed. The enhanced electrocatalytic performance and photovoltaic properties of the obtained materials are also provided, emphasizing the role of atomic substitution in achieving these advancements. Finally, challenges and future prospects in the field of atomic substitution for fabricating low-dimensional TMC materials are summarized.

## Highlights

- Atomic substitution applied in the synthesis of different dimensional transition metal chalcogenide (TMC) is dissertated.
- The controllable synthesis and property modification realization with atomic substitution or ion exchange are introduced.
- The substitution principle and mechanism in different TMCs are concluded.

## Graphical abstract

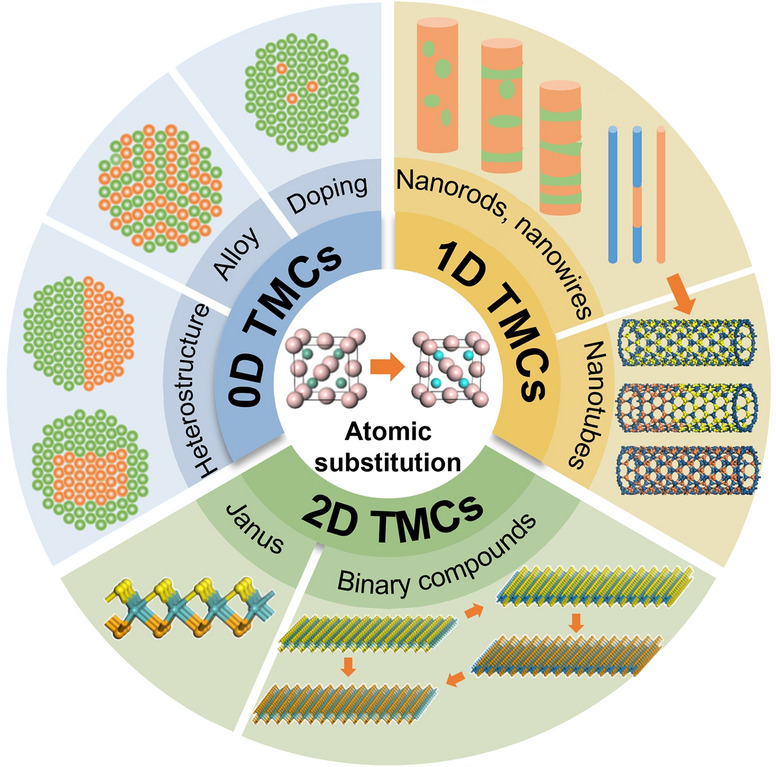

## Introduction

The diversity in types, structures, and properties of transition metal chalcogenides (TMCs) has led to their wide application prospects in devices, energy, and catalysis [1–3]. Especially those with reduced dimensions, including zero-dimensional (0D), one-dimensional (1D) and two-dimensional (2D) structures, have attracted significant attention due to their unique properties differing from their bulk counterparts [4–9]. To realize the applications of low-dimensional TMC materials in various scientific and technological fields, it is essential for these materials to exhibit well-defined optical, electronic, magnetic, and catalytic properties. Thereby, the synthesis methods that enable the precise control over the structures, morphologies and compositions of TMC materials are highly desired.

In the past few decades, different synthesis strategies for TMC materials have been developed, such as thermal injection and hydrothermal synthesis for 0D and 1D TMCs [10, 11], chemical vapor deposition (CVD) and molecular beam epitaxy (MBE) methods for 2D TMCs [12–15]. Although TMC materials with different sizes and morphologies have been realized, their qualities and compositions are generally limited [16, 17]. Due to the complex dynamics of reactions that involve multiple components, the controllability of direct synthesis methods is restricted. In contrast, atomic substitution shows great advantages in controlling the shapes, morphologies and compositions of multi-element materials. To date, the synthesis of various TMC structures have been realized by atomic substitution methods, including single binary compounds, doped materials, alloys, heterostructures and others [18–27].

As a post-processing technique, atomic substitution provides opportunities for precise engineering and customization of the desired properties of multi-element materials, and therefore serves to address the limitations of direct synthesis methods. By this method, great achievements have been made in TMCs synthesis and property modulation. For example, to achieve TMC materials with desired electronic or catalytic properties, ion exchange methods are commonly employed to tune the composition of the transition metal and chalcogens [28–33]. Moreover, the substitution reaction has the potential in inducing structural transformation, providing a promising pathway for synthesizing new materials or those are difficult to obtain directly [34–39]. For instance, through a moderate expansion of the anion sublattice during the substitution reaction process, it is possible to achieve metastable core@shell heterostructures without the formation of strain-induced defects at the interface, despite of a moderate lattice mismatch between core and shell [40]. These metastable heterostructures can hardly be directly synthesized by conventional growth methods, due to thermodynamically limited [41, 42]. Such controlled structural evolution not only expand the range of accessible materials but also enable the exploration for novel properties and functionalities, which is definitely advances the field of nanoscience.

The fast development of atomic substitution methods has made the precise synthesis of TMC materials with well-controlled properties possible. Here, we reviewed recent progresses in the synthesis and modulation of low-dimensional TMC materials, including 0D, 1D and 2D, achieved by atomic substitution methods. In the 0D section, the principles of atomic exchange in solution are discussed. The synthesis of substituted TMCs through both the cation exchange reactions and anion exchange reactions is presented. Moving to the 1D section, the synthesis of TMC nanorods, nanowires, nanotubes and nanobelts is summarized, highlighting the strategies employed to achieve their controlled growth. In the 2D section, the initiation mechanisms for atomic substitution within TMC films are introduced, which typically originate from vacancies, grain boundaries or edges. The substitution processes for 2D TMCs are discussed, categorized as either complete substitution or partial substitution, depending on the extent of the process. Complete substitution shows great potential in fabricating ultra-thin TMC films that cannot be directly synthesized, whereas partial substitution is widely employed for the synthesis of heterostructure materials, Janus structures, alloys and so on. The improved electrocatalytic and photovoltaic properties of the obtained TMC materials are also presented. Finally, we conclude this review by discussing the limitations and future. This comprehensive review provides valuable insights into the design principles, structural characteristics, and potential applications of low-dimensional TMC materials, which will greatly benefit the development of next-generation TMC devices and technologies.

## 0D TMCs

0D TMC materials refer to structures with all dimensions being in the range of 1 ~ 100 nm, which typically consist of transition metals from Group IB to Group IIB and chalcogens, with a composition ratio of M:X ranging from 1:1 to 1:2 [43–45]. Their unique physical and chemical properties make them to be highly promising for photoluminescence [46, 47], photocatalysis [48, 49], photothermal therapy [50, 51], etc. To date, many strategies have been developed to synthesize 0D TMC materials, such as hot injection [52, 53], topological chemical synthesis [54, 55] and solvothermal (or hydrothermal) method [56, 57]. Among them, the topological chemical synthesis approach accompanied with atomic substitution shows great advantages in precisely tailoring the compositions, morphologies and structures of materials [58–60]. It is worth noting that for 0D TMC materials, atomic substitute reactions typically take place within solutions containing ionic compounds, known as ion exchange reactions.

Since Alivisatos and coworkers demonstrated the potential of ion exchange in fabricating inorganic nanocrystals with diverse compositions, sizes, shapes and structures [61], this synthesis approach has gained widespread exploration for generating various materials. To date, ion exchange has become as a versatile tool for synthesizing TMC materials. In this section, the principles of ion exchange reactions were discussed, followed by the employment of ion exchange reactions in synthesizing various 0D TMC materials, including single-, doped-, alloyed- and hetero-nanocrystals.

### Principles of Ion Exchange Reactions

Generally, ion exchange reactions are reversible, with the reaction direction dominated by the thermodynamic factors of the reactants and products. Both the Gibbs free energy of reactions (Δ*G*_r_) and bond dissociation energies (BDEs) are essential for predicting the thermodynamics of ion exchange reaction [62, 63]. Δ*G*_r_ determines whether the reaction is thermodynamically favorable or not, and the reaction can proceed spontaneously when Δ*G*_r_ < 0. BDEs theory suggests that materials with stronger BDEs are more likely to participate in ion exchange reactions, especially when various ions are miscible. BDEs of some common metal chalcogenides are listed in Table 1. The synthesis of tellurides is more challenging compared to sulfides and selenides, owing to the lower BDEs between metal and Te atoms.

**Table 1:** Comparison of bond dissociation energies (BDEs) of some metal chalcogenides

Compound	BDEs (kJ mol^−1^)	Compound	BDEs (kJ mol^−1^)
Ag_2_S	216.7 ± 14.6	CdSe	127.6 ± 25.1
Ag_2_Se	210.0 ± 14.6	CdTe	100.0 ± 15.1
Ag_2_Te	195.8 ± 14.6	ZnO	280.1
Cu_2_O	259.0 ± 30.0	ZnS	224.8 ± 12.6
Cu_2_S	274.5 ± 14.6	ZnSe	170.7 ± 25.9
Cu_2_Se	255.2 ± 14.6	ZnTe	117.6 ± 18.0
Cu_2_Te	230.5 ± 14.6	PbS	398.0
CdO	236.0 ± 84.0	PbSe	302.9 ± 4.2
CdS	280.5 ± 20.9	PbTe	249.8 ± 10.5

**Fig. 1:**
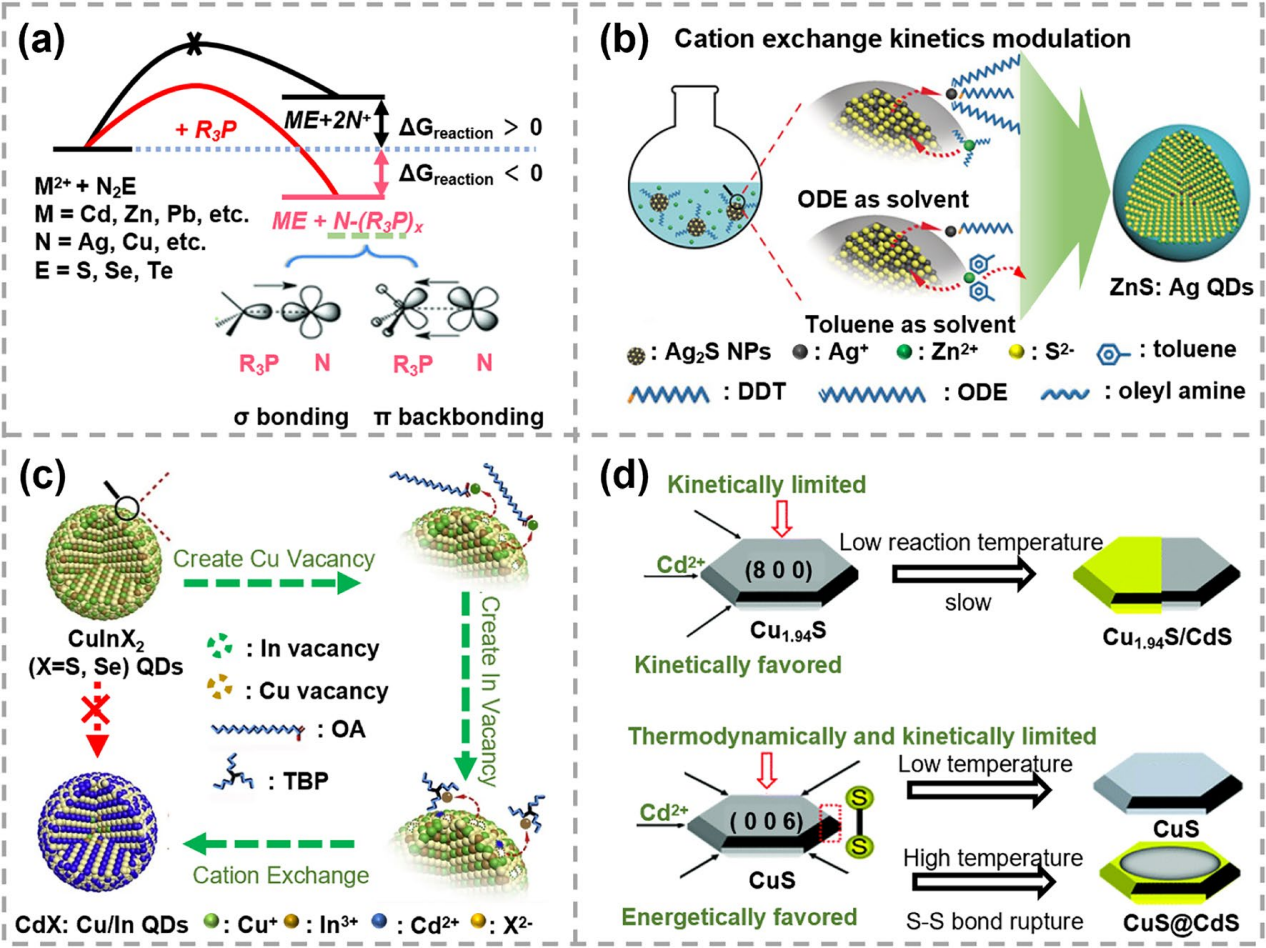
Schematics of ion exchange reaction principles **a** Thermodynamic scheme of the cation exchange (CE) reactions initiated by the phosphine ligands. Reproduced with permission [65]. Copyright 2015, John Wiley and Sons. **b** Schematic of the thiol ligands and solvents combination coordinated CE reactions. Reproduced with permission [66]. Copyright 2019, John Wiley and Sons. **c** Schematic of CE reaction triggered by surface vacancy engineering. Reproduced with permission [67]. Copyright 2020, Elsevier. **d** Scheme of partial CE reactions in djurleite Cu_1.94_S nanodisks and covellite CuS nanodisks. Reproduced with permission [68]. Copyright 2022, RSC Pub

The thermodynamics and kinetics of ion exchange reactions can also be effectively mediated by factors like ligands, solvents and crystal structures, etc., which are not taken into account in the above two theories. Currently, Pearson’s hard and soft acid base theory is widely used for assessing the affinity between metal ions and ligands/solvents [64]. It offers a framework to predict the process of ion exchange reactions, with hard acids showing a preference for hard bases and soft acids favoring soft bases. Phosphines as soft Lewis bases are common ligands in ion exchange reactions. Depending on the nature of the R groups, phosphines can form either σ bonding or π back-bonding with metal. In 2015, Zhang’s group intuitively characterized the distinct coordination capabilities between various metal cations and phosphine ligands, providing direct evidence for cation exchange (CE) reactions induced by phosphine ligands [65]. As shown in Fig. 1a, for a thermodynamically unfavorable exchange process (Δ*G*_r_ > 0), the introduction of phosphine ligands can not only facilitate the solvation and extraction of cations from the reactants but also enhance the desolvation and incorporation of cations into the products. This effectively changed the sign of Δ*G*_r_ (Δ*G*_r_ < 0), making the reaction thermodynamically favorable. Based on this finding, a series of metal@semiconductor core@shell nanocrystals were successfully obtained by choosing appropriate phosphine ligands. Solvents also play a crucial role in determining the kinetics of ion exchange reaction. As illustrated in Fig. 1b, Bai et al. synthesized high-quality Ag-doped ZnS quantum dots (QDs) and Au@ZnS core–shell nanocrystals using different solvents [66]. It was found that even utilizing the same thiol ligand, CE reactions in different solvents shows distinct reaction rates, suggesting diverse coordination abilities between metal cations and solvents.

The influence of defects and crystal structures of reactants on the process of ion exchange reaction cannot be ignored. Vacancy defects act as vehicles to promote ion diffusion as they can move quickly within the crystal during reactions. As illustrated in Fig. 1c, using engineered CuInX_2_ nanocrystals with abundant surface vacancies as the reactant, Bai et al. successfully synthesized Cu/In dual-doped CdX (*X* = S, Se) nanocrystals [67]. The creation of Cu and In vacancies are found to be critical to overcoming kinetic energy barriers and effectively accelerating Cd^2+^ diffusion process. As for the crystal structure, it mainly affects the diffusion of ion into host materials. In Fig. 1d, due to the similar structures between djurleite Cu_1.94_S and wurtzite CdS, Cu^+^ in djurleite Cu_1.94_S nanodisks can be easily substituted by Cd^2+^ at a low temperature [68]. It is worth noting that the CE reactions from the top of Cu_1.94_S nanodisks were kinetically limited due to the absence of vacancies along the < 100 > direction, resulting in the formation of segmented Cu_1.94_S/CdS nanodisks. In contrast, Cu^2+^ in covellite CuS were hardly replaced by Cd^2+^ at a low temperature, due to the high energy barrier for breaking S–S covalent bonds. At a high temperature, S–S bonds underwent reduction, leading to the reorganization of sulfur anion frameworks. As a result, the substitution of Cu^2+^ in CuS by Cd^2+^ along the lateral directions became energetically favorable. However, CE reactions starting from the top of the CuS nanodisks were limited because of the large mismatch of S–S distances between CuS (001) and CdS (001), resulting in the formation of CuS@CdS nanodisks.

### Complete Exchange

To date, the ion exchange strategy has been widely used for controllable synthesis of TMC materials with tailored morphologies, structures and hetero-interfaces. Especially, it allows synthesis of TMC materials with unique compositions and morphologies that cannot be obtained by direct seeded growth. For example, metal@semiconductor core@shell heterostructures through ions exchange of semiconductor shells with guest ions showed atomically organized interfaces and high-crystalline semiconductor shells, and such structures can be achieved even there is a large lattice mismatch between metal and semiconductor components [65, 69–71]. These structures generally enable efficient injection of hot electrons triggered by surface plasmon resonance, which provides an avenue to promoting the efficiency of photocatalysis, photoelectrochemical cells and photovoltaics.

#### *Syntheses of 0D TMCs *via* CE Reactions*

**Fig. 2:**
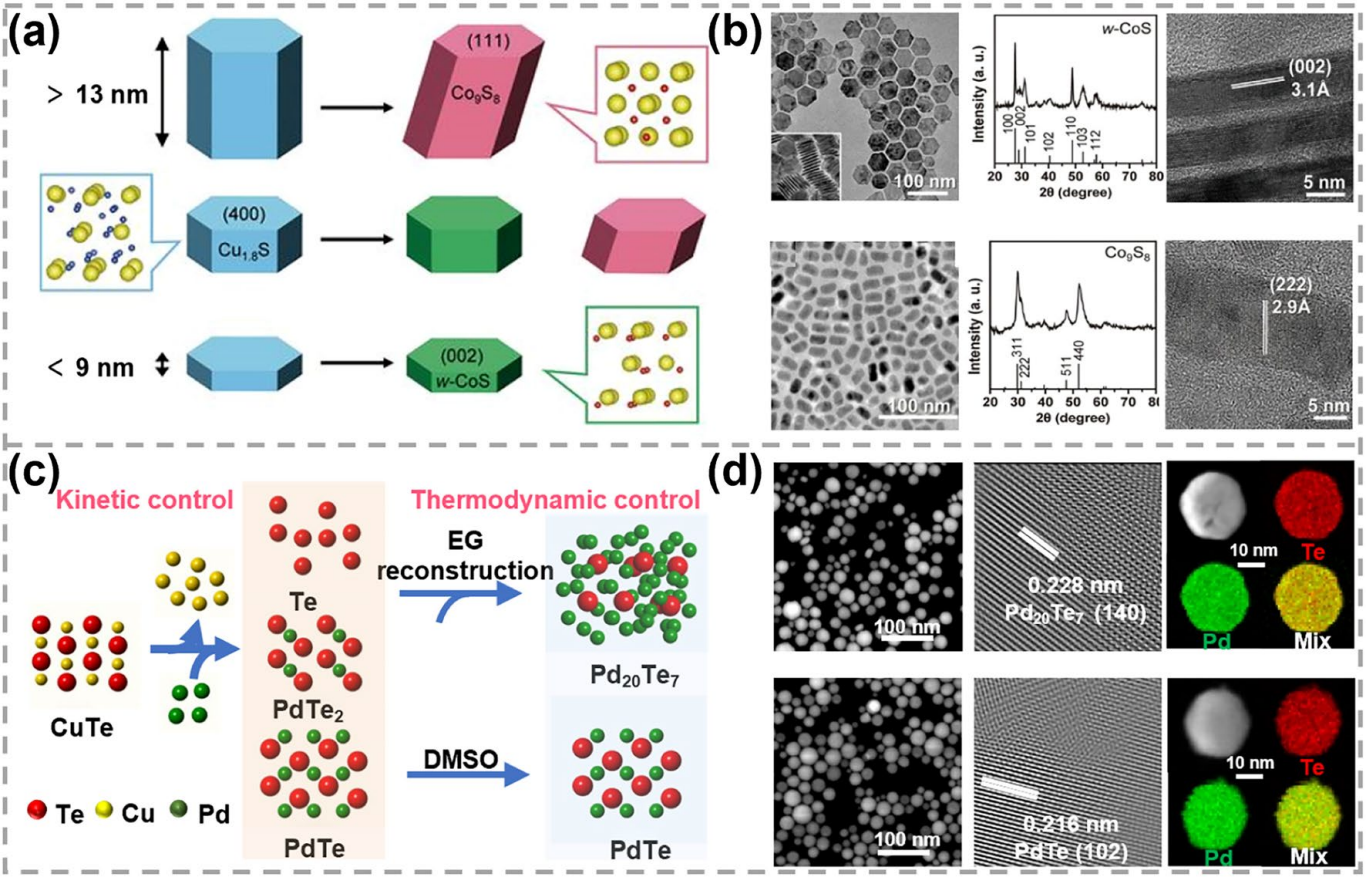
Synthesis and characterizations of 0D TMC materials obtained by CE reactions. **a** Schematics of height-dependent phase transformation of Cu_1.8_S nanocrystals during the CE process and **b** corresponding transmission electron microscopy (TEM) images, X-ray diffraction (XRD) patterns, high-resolution transmission electron microscopy (HRTEM) images. Reproduced with permission [38]. Copyright 2021, The American Association for the Advancement of Science. **c** Schematic showing the kinetic control and thermodynamic control for the CE process of PdTe and Pd_20_Te_7_ nanospheres synthesis. EG represents ethylene glycol and DMSO represents dimethyl sulfoxide. **d** TEM, HRTEM images of PdTe_2_ and Pd_20_Te_7_ nanospheres and corresponding energy-dispersive X-ray spectroscopy (EDS) elemental mapping images. Reproduced with permission [60]. Copyright 2022, Springer Nature

According to the type of exchanged element, ion exchange reactions can be categorized into the CE reactions and anion exchange (AE) reactions. CE reactions involve the release of cations from the reactant TMC material and the exchange of other cations from the solution into TMC material. After CE reactions, the anion framework can be either preserved or reconstructed [63, 72–74]. As shown in Fig. 2a, b, the original S framework can be maintained when the height of Cu_1.8_S nanocrystals was less than 9 nm, resulting in the formation of wurtzite cobalt sulfide (CoS) [38]. Once the height exceeds 13 nm, structure reconstruction driven by thermodynamics occurred and cubic cobalt pentlandite (Co_9_S_8_) nanocrystals was obtained.

CE reactions have been widely used for the controllable synthesis of M–S and M-Se materials [55, 75–80]. However, the transformation of M–Te materials is often accompanied with significant alteration in morphology, making the synthesis of M–Te materials with desired morphologies and structures to be more challenging [63, 81]. Recently, Feng et al. synthesized noble metal tellurides (NMTs) with tailored morphologies (0D, 1D, 2D and 3D), compositions (Pd/Pt/Rh/Ru/Ag/Au-based NMTs) and structures via CE strategy by regulating the interactions between solvents and noble metal cations [60]. As displayed in Fig. 2c, Cu^2+^ within the CuTe template can be rapidly exchanged by Pd^2+^ based on the kinetic control, leading to the formation of Te, PdTe_2_ and PdTe. The products can be further tuned by controlling the thermodynamic process. In dimethyl sulfoxide, Pd^2+^ formed strong bound with the sulfoxide group, leading to a low content of free Pd^2+^ in solution for CE reactions. This resulted in the formation of Pd-poor NMTs, specifically PdTe. In contrast, there were more Pd^2+^ cations available for CE reactions in ethylene glycol due to its much weaker interaction with Pd^2+^. As a result, a Pd-rich structure characterized as Pd_20_Te_7_ was formed. Despite of the reconstruction of anion framework, the original nanosphere shape was retained in Pd_20_Te_7_ and PdTe products, showing highly crystalline structures with a uniform element distribution (Fig. 2d). In this strategy, the kinetic control and solvent-dependent thermodynamic control were revealed to be critical for the successful synthesis. This CE reaction provide a feasible and flexible method for the achieving of desired NMTs.

**Fig. 3:**
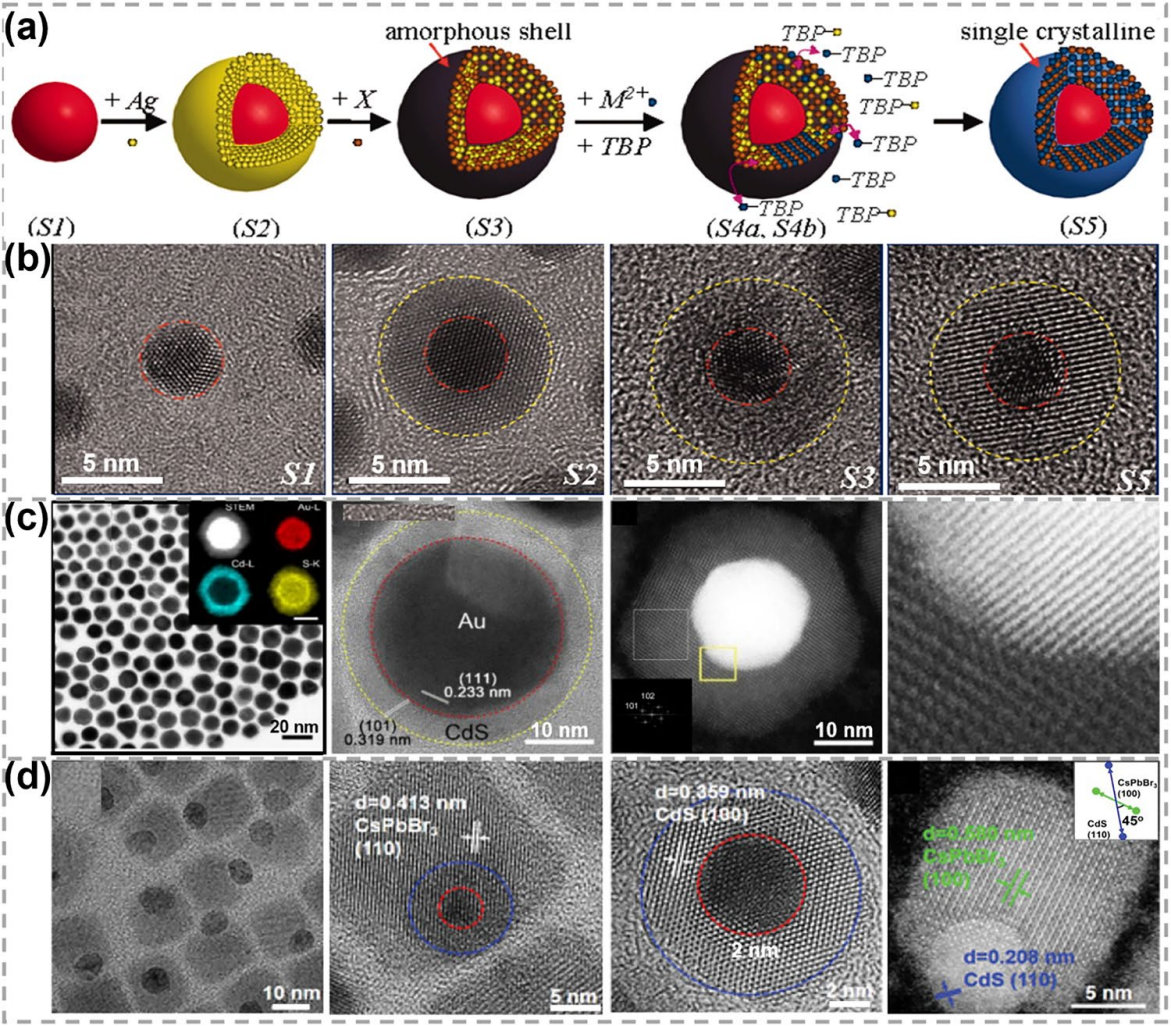
Synthesis and characterizations of 0D metal–semiconductor heterostructures obtained by the reverse CE reaction-facilitated non-epitaxial growth strategy. **a, b** Schematic showing different growth stages of Au@CdS heterostructures and the corresponding HRTEM images. Reproduced with permission [69]. Copyright 2010, The American Association for the Advancement of Science. **c** TEM images of Au@ CdS heterostructures and high-angle annular dark-field scanning TEM (HAADF-STEM) images with corresponding elemental mapping images. Reproduced with permission [48]. Copyright 2018, Elsevier. **d** TEM and HAADF-STEM images of Au@CdS/CsPbBr_3_ heterostructures. Reproduced with permission [71]. Copyright 2022, John Wiley and Sons

In addition to the synthesis of pure phase of TMCs, CE reactions also provide a promising strategy for fabricating metal–semiconductor heterostructures. In Table 2, representative examples of such heterostructures with varying lattice mismatches are listed. Notably, CE reactions demonstrate a great potential in synthesizing heterostructures with substantial lattice mismatches, which is typically beyond the capability of conventional epitaxial growth methods. In 2010, Zhang et al. reported a synthetic route for metal@semiconductor core@shell heterostructures [69], as exhibited in Fig. 3a, b. Starting from the metal core, an amorphous shell of Ag_2_X (*X* = S, Se, Te) was obtained first, serving as a crucial platform for the following reverse CE reactions. Tributylphosphine was then selected as the phase-transfer agent to exchange free cations (M^n+^) in solution with Ag^+^ in the amorphous matrix, in order to obtain Au@MX (*X* = S, Se, Te) heterostructures. Various core@shell heterostructures were achieved finally, including Au-CdSe, Au-CdTe, FePt-CdS, Au-PbS, Au-ZnS and Pt-CdS. These heterostructures all possessed atomically organized interfaces and monocrystalline shells. The lattice structures of TMC shells were found to be independent of the core metal, suggesting their non-epitaxial relationship. With the development of this reverse CE reaction-facilitated non-epitaxial growth strategy, the size of synthesized high-quality metal@semiconductor heterostructures had reached to 50–100 nm [48], achieving breakthroughs from quantum scale to nanoscale (Fig. 3c). In these studies, the shell growth was guided by its thermodynamic property, and the obtained structure was independent of the core. The CE reaction is beneficial for circumventing the limitations imposed by epitaxial strategies and offers a precise and controllable way in the synthesis of core@shell heterostructures with atomically organized interfaces.

**Table 2:** Representative examples of reported core@shell heterostructures with different lattice mismatches

Core	Shell	Lattice mismatches (%)	Overall morphology	Refs.
PbTe	CdTe	< 1	Sphere	[29]
PbSe	CdSe	~ 1	Sphere/rod/cube	[82, 83]
PbS	CdS	~ 2	Rod/sphere	[84, 85]
CuInS_2_	ZnS	2–3	Sphere	[86]
CdSe	CdS	3.8–3.9	Rod/sphere/tetrapod	[87, 88]
CdTe	ZnTe	6.5	Sphere	[89]
CdTe	CdSe	6.7–7.1	Sphere/prolate-shape	[90, 91]
CdTe	ZnS	19.8	Sphere	[89]
Ag	CdS	35.3	Triangle	[92]
Au	CdS	42.7	Sphere	[69]
Pt	CdS	48.3	Sphere/cube	[65, 69]
Au	CdSe	49.1	Sphere/dumbbell	[69, 93]
Au	CdTe	58.9	Sphere/rod	[69, 94]

This non-epitaxial growth strategy also shows a great potential in manipulating the compositions and structures of heterostructures [71, 95]. For instance, by maneuvring interfacial strain between metal and semiconductor components, non-concentric Au@CdX heterodimer structures were achieved [70]. The tunable relocations of plasmonic Au to CdX (X = S, Se, Te) in quantum size region allowed for a high degree of tunability in coupling their optoelectronic properties with other structures. By a combined non-epitaxial/epitaxial strategy, Au@CdS/CsPbBr_3_ ternary heterostructures with atomically organized interfaces were obtained (Fig. 3d). These resulting Au@CdS/CsPbBr_3_ heteronanocrystals generated remarkably long-lived plasmon-induced charge carriers with lifetime up to nanosecond timescale [71]. This development further expanded the applications of the reverse CE reaction-facilitated non-epitaxial growth strategy.

#### *Syntheses of 0D TMCs *via* AE Reactions*

**Fig. 4:**
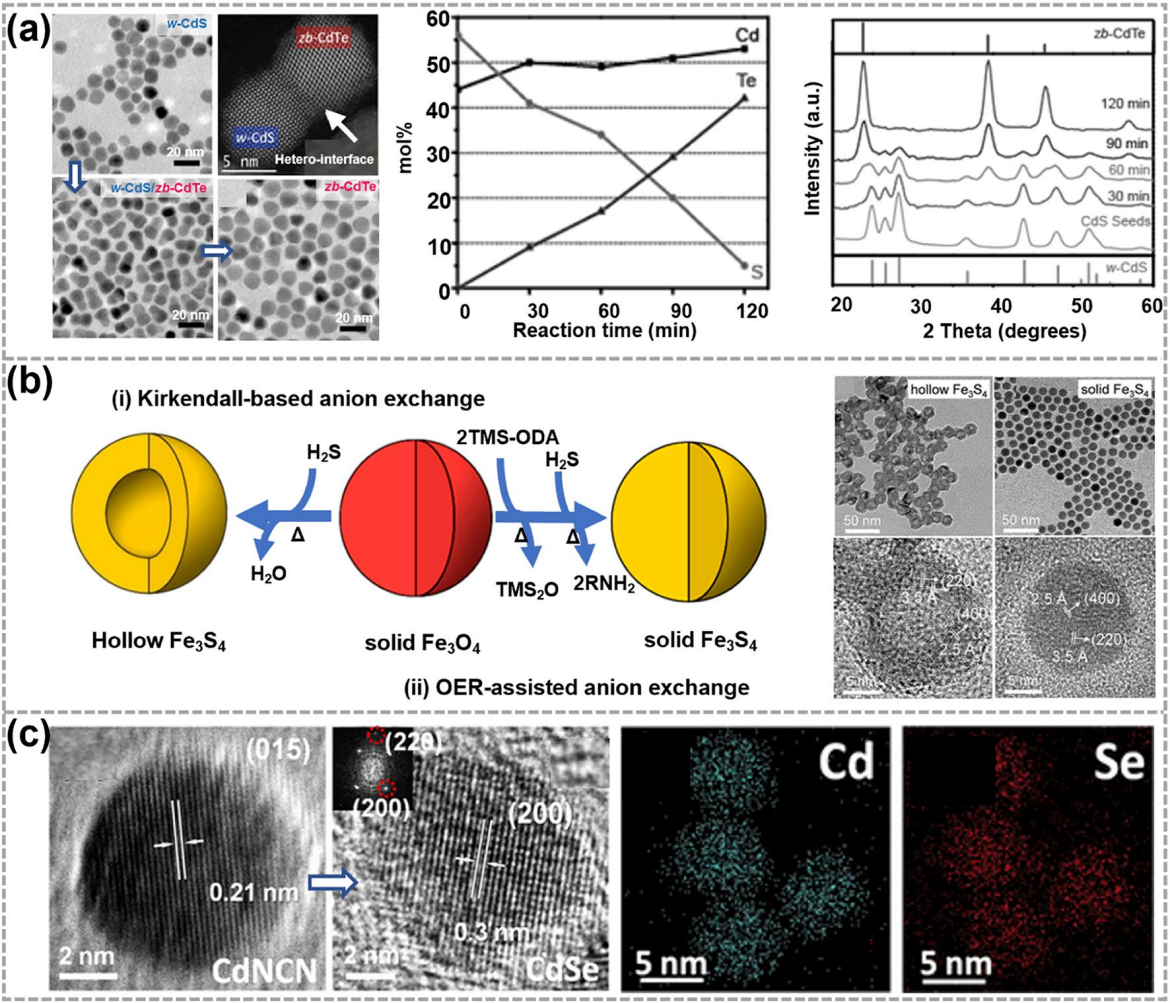
Synthesis and characterizations of 0D TMC materials obtained by the anion exchange (AE) reactions. **a** Synthesis illustrations of *w*-CdS/*zb*-CdTe heterodimers and corresponding TEM and HAADF-STEM images. Reproduced with permission [96]. Copyright 2011, American Chemical Society. **b** Illustrations showing the synthesis of hollow Fe_3_S_4_ and solid Fe_3_S_4_ nanocrystals and corresponding TEM and HRTEM images. OER represents an oxygen extracting reagent, TMS-ODA represents (Z)-N-trimethylsilyloctadec-9-en-1-amine and TMS_2_O represents bis-(trimethylsilyl) oxide. Reproduced with permission [97]. Copyright 2020, American Chemical Society. **c** Illustrations showing the synthesis of MX nanoparticles (*M* = Cd, Mn; *X* = S, Se) and corresponding HRTEM image as well as EDS elemental mapping images. Reproduced with permission [98]. Copyright 2019, American Chemical Society

The process of AE reactions refers to exchanging anions in the reactant material with other anions. Compared with cations, the diffusion of anions is much slower within the 0D TMC nanocrystals due to their large ionic radii and large diffusion barrier. Consequently, AE reactions usually require higher reaction temperatures and longer reaction times, which contributes to a more manageable and controllable reaction process. As displayed in Fig. 4a, by performing the AE reactions of S^2−^ by Te^2−^ at 260 °C, the wurtzite CdS nanocrystals can be gradually transformed into zinc blende CdTe [96]. During the AE process, the size of the CdTe phase increased gradually, and spontaneous phase segregation occurred between CdS and CdTe due to their different phase structures. As the reaction continued, the complete transformation to a high-crystalline zinc blende CdTe was achieved at 120 min.

Besides, AE reactions not only occur between chalcogens but can also take place between other elements. As shown in Fig. 4b, Lim et al. synthesized morphology-conserving Fe_3_S_4_ nanocrystals by AE reactions with the aid of oxygen extracting reagents [97]. In the AE process, the extraction of oxygen and subsequent formation of an amorphous phase acted as accelerants for the diffusion of incoming S^2−^ ions, leading to the formation of morphology-conserving Fe_3_S_4_ nanocrystals (Fig. 4bii). Conversely, in the absence of oxygen-extraction agents, the formation of hollow Fe_3_S_4_ nanocrystals was inevitable, due to the imbalance in the diffusion between outgoing and incoming ions (Fig. 4bi). The oxygen extracting reagents assistant method has a great advantage in the synthesis of designed 0D nanocrystals without voids. This strategy illustrated new possibilities for the synthesis of various TMCs regardless of their shape and crystal structure. Jia et al. found that quasi-linear NCN^2−^ in MNCN (*M* = Cd, Mn) nanoparticles can also be exchanged by X^2−^ (*X* = S, Se) in colloidal solution (Fig. 4c). The quasi-linear NCN^2−^ generated open space within the crystal lattice, effectively accommodating internal stress induced by AE reactions. As a result, the morphology and crystallinity were retained throughout the AE process. Besides, the anisotropic lattice of [NCN]^2−^ anions was revealed to be critical in avoiding diffusion rate disparities and lattice collapse that occurred in single-atom anion compounds [98].

### Partial Exchange

Compared with the complete exchange, partial anions/cations exchange process shows great advantages to synthesize doped, alloyed, and heterostructure TMC materials [10, 66, 99–104]. These materials have been widely reported due to their enhanced properties and optoelectronic applications [105–107]. For example, the deep-site doping enabled by reverse CE reactions can lead to nanocrystals with improved fluorescence quantum yields and lifetimes [100]. Core@shell QDs with continuously graded semiconductor shells prepared by ion exchange reactions show a strain-free interface and well-confined exciton, which are promising in optoelectronic applications of luminescent solar concentrators, light emitting devices and laser [108, 109]. Moreover, the ion exchange reaction provides possibilities for achieving metastable or non-equilibrium nanostructures, which cannot be obtained by conventional hot injection synthesis [42, 110]. In this section, we discuss the status of partial exchange in preparing doped, alloyed and hetero-structured TMC materials. In general, alloyed and doped materials were obtained in the case of reactants and products were miscible; otherwise, multi-domain heterostructures were formed.

#### Synthesis of 0D Doped TMC Materials

Due to the inherent self-purification effects, the host matrix often exhibits a tendency to expel dopant ions toward the surface of nanocrystals during doping process, especially at high temperatures [100]. Therefore, a delicate balance in precursor reactivity is required for precise control over the incorporation of dopants, which is quite challenging in a direct synthesis process. In contrast, the ion exchange strategy shows great advantages in the synthesis of doped materials, as it allows for the temporal separation of nanocrystal growth from the incorporation of impurities [63]. By controlling the kinetics of partial CE process, it is possible to retain a small quantity of “impurities” within the host matrix, serving as dopant ions. To date, various 0D doped TMC nanocrystals have been reported by direct CE reactions, such as Yb-doped PbIn_2_S_4_ nanocrystals [111], Ag-doped CdSe nanoparticles [112], Mn-doped ZnSe QDs [113] and Cu- or Ag-doped Cd_1-x_Zn_x_Se nanocrystals [114]. All of these materials exhibited a much broader tunable spectrum range and enhanced optoelectronic properties, depending on the concentration of dopants.

**Fig. 5:**
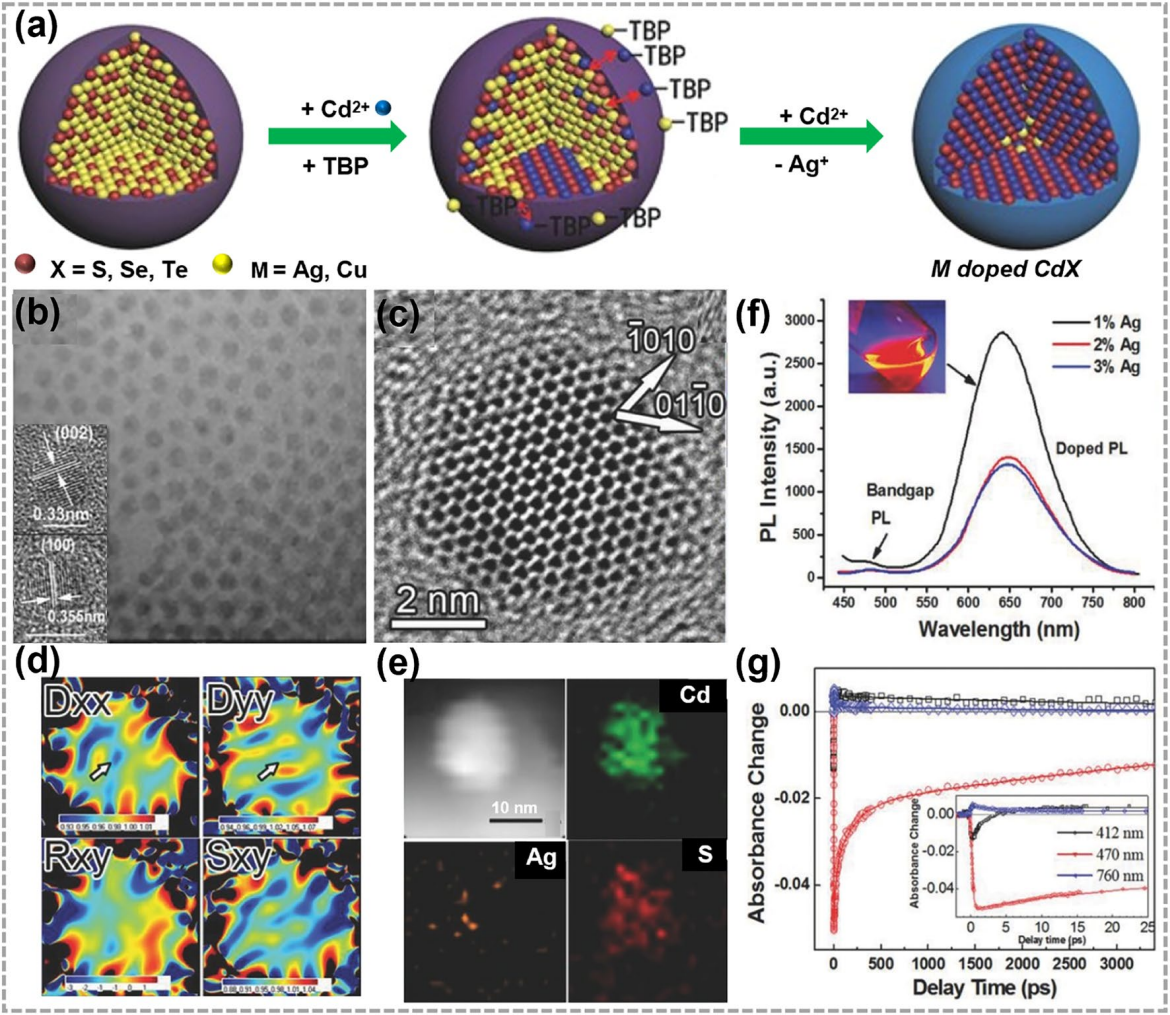
Synthesis and characterizations of 0D doped TMC materials by CE reactions. **a** Illustrations showing the synthesis process of M-doped CdX (*M* = Ag, Cu;* X* = S, Se, Te) nanocrystals. TBP represents tributylphosphine. **b** TEM image, **c** HRTEM images, **d** strain mapping images and **e** EDS elemental mapping images of Ag-doped CdS quantum dots (QDs). **f** Room temperature steady-state fluorescence spectra with different Ag-dopant concentrations, and the inset shows a digital photograph of fluorescence under 365 nm ultraviolet irradiation. PL represents photoluminescence. **g** Kinetic traces at representative wavelengths are also shown (pump laser wavelength: 390 nm). Reproduced with permission [100]. Copyright 2015, John Wiley and Sons

Besides, as shown in Fig. 5a, Zhang et al. synthesized M-doped CdX (*M* = Ag, Cu and *X* = S, Se, Te) QDs by reverse CE reactions. By controlling CE process of M^+^ and Cd^2+^, the deep-site heterovalent doping can be achieved. Transmission electron microscopy (TEM) and high-resolution transmission electron microscopy (HRTEM) images in Fig. 5b, c show that the obtained QDs were monodispersed and highly crystalline. Figure 5d, e confirms that Ag atoms were effectively retained within the center of CdS QDs, suggesting the realization of deep-site heterovalent doping rather than a surface doping [100]. Deep-site heterovalent doping effectively avoided the self-purification process. The efficient energy transfer from the intrinsic conduction band to the deep dopant energy level quenched exciton emission and inspired stable and strong dopant emission (Fig. 5f, g). This synthesis paves the way for the incorporation of dopant ions into specific sites of nanocrystals.

#### Synthesis of 0D TMC Alloys

**Fig. 6:**
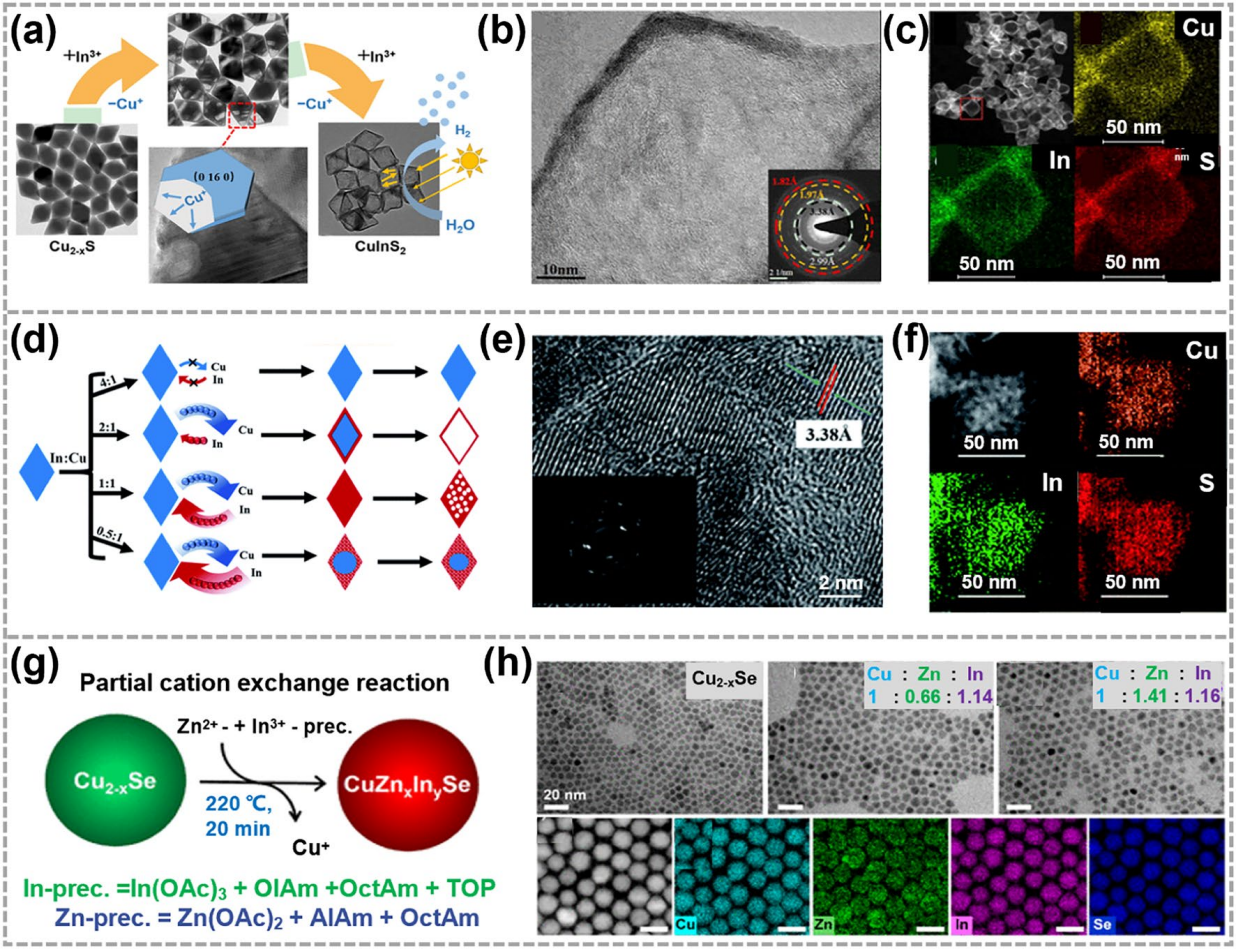
Synthesis and characterizations of TMC alloys by CE reactions. **a** TEM images showing the structural evolution from Cu_2-x_S dodecahedrons to CuInS_2_ ones. **b, c** STEM images and corresponding EDS elemental mapping images of CuInS_2_ dodecahedrons. Reproduced with permission [18]. Copyright 2019, American Chemical Society. **d** The different products derived from In^3+^—for—Cu^+^ CE in Cu_7_S_4_ nanocrystals at different In: Cu ratio. **e, f** HRTEM image and the corresponding EDS elemental mapping images for the product in d with the In: Cu ratio of 1: 1. Reproduced with permission [116]. Copyright 2019, Royal Society of Chemistry. **g** Illustrations showing the synthesis of CuZn_x_In_y_Se nanocrystals. **h** HRTEM images and corresponding EDS elemental mapping images of CuZn_x_In_y_Se nanocrystals. Reproduced with permission [118]. Copyright 2023, American Chemical Society

In direct synthesis of alloyed TMC materials, a significant challenge arises from the necessity of balancing the reactivities of precursors, which makes it difficult to attain the desired atomic composition. However, the development of CE reactions provides a feasible approach to overcome this challenge. Based on this strategy, binary, ternary and even quaternary alloyed nanocrystals have been realized [18, 106, 115–118]. As exhibited in Fig. 6a–c, Li et al. synthesized hollow CuInS_2_ nano-dodecahedrons with a uniform elemental distribution by using Cu_2-x_S as reactants. The formation of hollow structure was attributed to different rates of Cu^+^ extraction from Cu_2-x_S nanocrystals and In^3+^ incorporation into them [18]. The diffusion kinetics of Cu^+^ and In^3+^ can be precisely manipulated by passivating the surface of Cu_7_S_4_ nanocrystals. As shown in Fig. 6d–f, with an increased In^3+^/Cu^+^ ratio, the surface of Cu_7_S_4_ nanocrystals were passivated due to excess In^3+^ cations. As a result, a series of CuInS_2_ and Cu_7_S_4_@CuInS_2_ nanocrystals with intricate structures and uniform elemental distribution were obtained, which showed different photocatalytic abilities in singlet oxygen generation [116]. This research not only provides a flexible way to achieve nanocrystals with structural complexity and diversity, but also have an important significance in understanding the reaction kinetics in ion exchange process.

In 2023, Thiel et al. used Cu_2-x_Se nanocrystals as templates to synthesize CuZn_x_In_y_Se quaternary alloyed nanocrystals (Fig. 6g). HRTEM images and corresponding energy-dispersive X-ray spectroscopy (EDS) elemental mapping images demonstrated the complete transformation of Cu_2-x_Se nanocrystals into CuZn_x_In_y_Se nanocrystals, while maintaining high uniformity in the distribution of Cu, Zn, In and Se elements (Fig. 6h). The photoluminescence test results showed that the synthesized CuZn_x_In_y_Se nanocrystals exhibited excellent near-infrared photoluminescence, without the need for additional shell growth. This is notable because the most of developed protocols require an additional shell to achieve similar performance. In particular, the full width at half-maximum of their near-infrared photoluminescence was reduced to 150–190 meV, accompanied by an enhancement of photoluminescence quantum yield to 20%. Moreover, precise control over the In/Zn ratio was achieved, and it was observed that increasing the Zn content resulted in an observable blue shift [118]. Up to now, various 0D alloyed TMC materials have been realized by CE reactions, including Zn_x_Cd_13-x_Se_13_ magic-size clusters [117], AgInS_2_ nanoparticles [119] and Au@Ag_2_ZnSnS_4_ core–shell nanocrystals [120], etc. This synthesis strategy not only offers a protocol for the precise construction of 0D TMC nanocrystals with desired structures and composition, but also contributes to enhancing the structural complexity and diversity to TMC materials.

#### Synthesis of 0D TMC Heterostructures

TMC heterostructures have attracted lots of attention due to their capacity to integrate diverse functions stemming from coupling between multiple components**.** In recent years, partial CE reactions have emerged as a valuable alternative to epitaxial growth for the synthesis of TMC heterostructures at relatively low temperatures [121]. It also shows great advantages in controlling the interface between two components. To date, both segmented heterostructures and core@shell heterostructures have been realized by partial CE reactions. In segmented heterostructures, different materials are arranged in distinct segments or regions, including Janus-like, striped and sandwich-like structures.

**Fig. 7:**
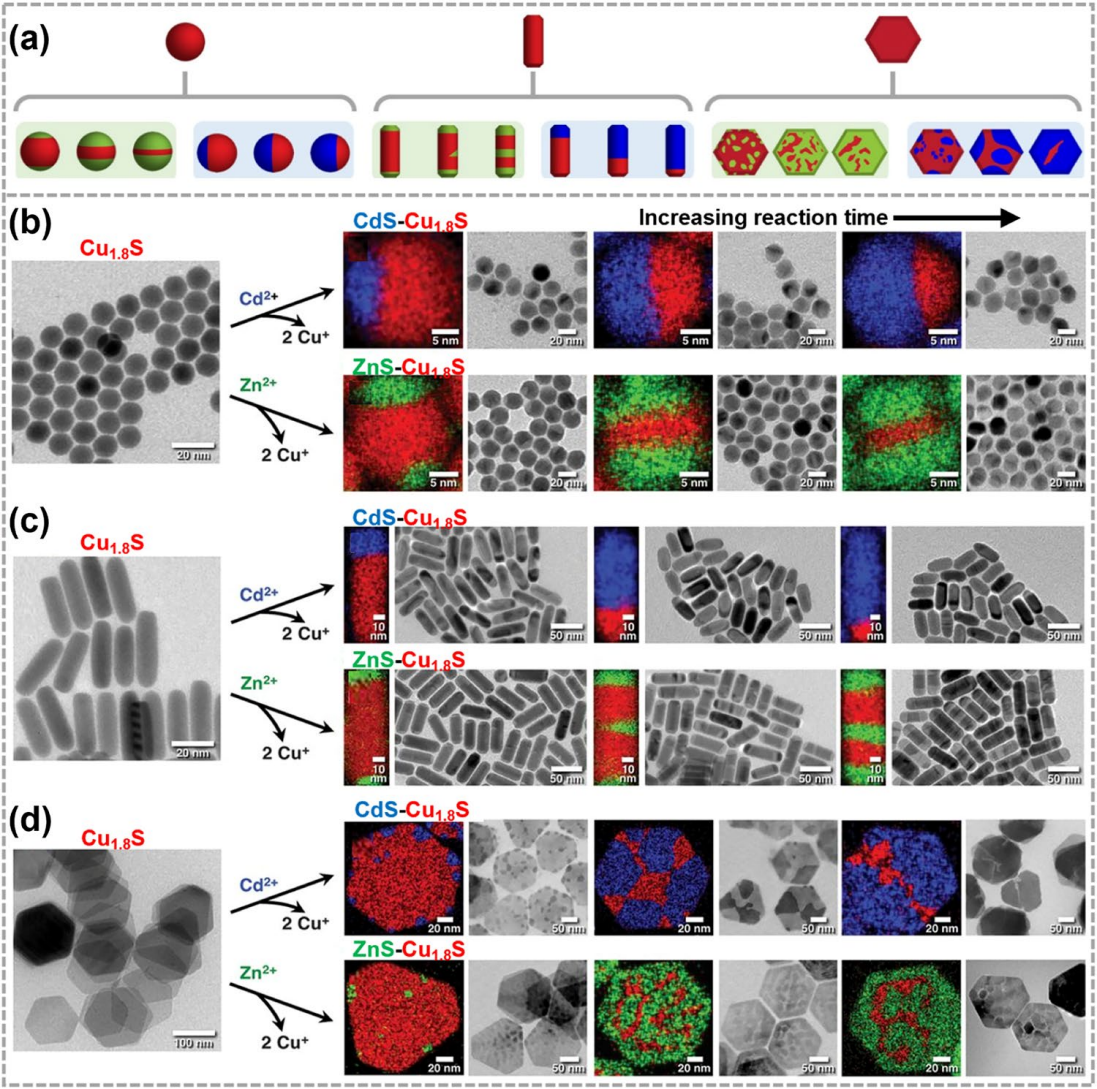
Synthesis and characterizations of the segmented heterostructures obtained by CE reactions.** a** Synthesis illustrations of Cu_1.8_S/CdS and Cu_1.8_S/ZnS segmented heterostructures. Reproduced with permission [122]. Copyright 2020, American Chemical Society. TEM images and corresponding EDS elemental mapping images of Cu_1.8_S **b** spheres, **c** rods and **d** hexagonal plates. Reproduced with permission [25]. Copyright 2018, The American Association for the Advancement of Science

For the synthesis of segmented heterostructures, the ion mobility is usually high enough to facilitate the atomic arrangement of transformed domains into the lowest energy configuration. For instance, due to the high mobility of Cu ions, Cu_2-x_S reactants have been extensively employed for producing Cu_2-x_S/MS (*M* = Cd, Zn, Pb) segmented heterostructures. In Fig. 7a, Fenton et al. used different shaped Cu_1.8_S nanoparticles as templates in CE process. By adjusting reaction time, the extent of CE reactions involving Cu^+^ with Cd^2+^ or Zn^2+^ can be controlled [122]. As a result, various types of Cu_1.8_S/CdS or Cu_1.8_S/ZnS heterostructures were achieved successfully, including particles that contain asymmetric, patchy, porous, and sculpted nanoarchitectures. These heterostructures exhibited clear segmentation pattern and distinct internal interfaces (Fig. 7b–d), as confirmed by TEM images and corresponding EDS elemental mapping images [25]. This modular and divergent synthetic strategy enables the design and synthesis of complex nanoparticle systems. These researches have illustrated the reaction time is essential for the control of heterostructure with different morphology. These intricate colloidal nanoarchitectures have diverse potential applications, such as semiconductor–semiconductor interfaces for the controllable separation or confinement of excitons, precision integration of semiconductors and catalysts for light-driven chemical transformations, etc.

**Fig. 8:**
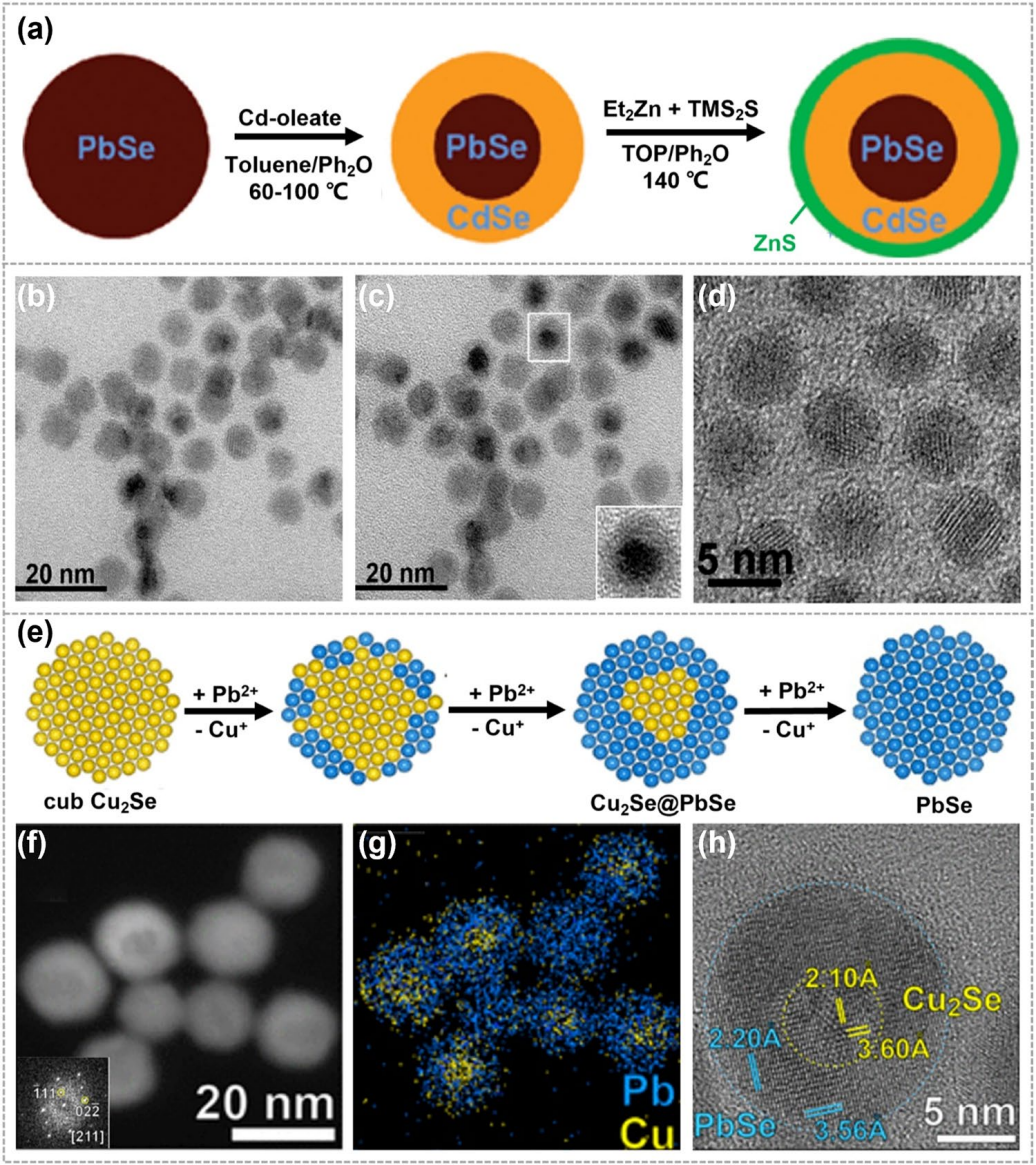
Synthesis and characterizations of the core–shell heterostructures obtained by CE reactions. **a** Synthesis illustrations of PbSe@CdSe QDs. Ph_2_O represents phenyl ether, TOP represents trioctylphosphine, Et_2_Zn represents diethyl zinc and TMS_2_S represents hexamethyldisilathiane. **b** Low-resolution image of 6.1 nm PbSe cores with 1.4 nm CdSe shells. **c** Low-resolution image of the same area as **b**, but tilted by 12° to enhance the diffraction contrast. **d** High-resolution image of PbSe@CdSe QDs, showing a relatively defect-free interface. Reproduced with permission [123]. Copyright 2008, American Chemical Society. **e** Cu_2_Se@PbSe heterostructures and **f–h** corresponding HRTEM images as well as corresponding EDS elemental mapping images. Reproduced with permission [40]. Copyright 2017, American Chemical Society

During the synthesis of core@shell heterostructures, CE reactions follow an isotropic-like exchange, without the generation of a significant strain between the two components. One of the extensively studied examples is PbX@CdX (*X* = S, Se, Te) core–shell heterostructures, where the core and shell have immiscible crystalline structures and a slight lattice mismatch. In 2008, Pietryga et al. applied CE reactions to prepare PbX@CdX QDs. As shown in Fig. 8a, PbSe QDs were exposed to a Cd-containing precursor, the partial substitution of Pb^2+^ by Cd^2+^ resulted in the formation of PbSe@CdSe QDs. The small lattice mismatch (∼1%) between the rock-salt PbSe and zinc-blende CdSe led to a relatively defect-free interface (Fig. 8b–d). The obtained PbSe@CdSe core@shell QDs exhibited significantly enhanced stability, thereby extending their operational temperature range [123]. In 2012, applying the similar strategy to PbSe nanocrystals of star, cube, and rod shapes, Casavola et al. achieved PbSe@CdSe core–shell heterostructures with corresponding shapes [83]. It was revealed that CE reactions proceeded dominantly in the (111) crystallographic direction, regardless of the shape of the initial PbSe QDs. This facilitated the formation of the Pb(111)—Se(111)—Cd(111) sandwich structure, resulting in a stress-free interface. Moreover, Pietryga et al. reported that a higher reaction temperature resulted in a thicker shell [124]. The thickness of CdSe shell up to 4 nm was achieved at a temperature of 200 °C, which displayed an additional infrared emission channel aside from the common visible range. The obtained heterostructures are a unique class of tuneable, dual-emitting, dispersible fluorophores, holding great potentials for labeling and photoluminescence microscopy. The critical factor of the core@shell heterostructure formation is to overcome the strain at interface resulting from lattice mismatch.

Precise control over the kinetics of CE reactions allows for the synthesis of core–shell heterostructures with a moderate lattice mismatch. However, these obtained core–shell heterostructures are usually metastable and prone to transform into more stable configurations, especially under conditions of elevated temperatures or intense light irradiation [80, 125–127]. For example, by performing partial Cu^+^ ions exchange with Pb^2+^ or Sn^2+^ within Cu_2-x_Te nanocubes, metastable Cu_2-x_Te@PbTe or Cu_2-x_Te@SnTe heterostructures were synthesized. The rapid and non-selective CE reactions took place on all facets of Cu_2-x_Te nanocubes, owing to abundant vacancies on the surface and high diffusion rate of Cu^+^ ions. Due to the significant deviation in lattice constants from that of Cu_2-x_Te, the formed PbTe or SnTe shells were polycrystalline. These metastable core@shell heterostructures transformed into stable Janus-like heterostructures when annealed at 200 °C in vacuum [42]. Additionally, it is possible to achieve core@shell heterostructures without the formation of strain-induced defects at the interface through a moderate expansion of the anion sublattice, even when there is a moderate lattice mismatch between core and shell. As shown in Fig. 8e, cubic Cu_2_Se nanocrystals were employed as host materials in CE reactions, and Cu_2_Se@PbSe heterostructures with a small expansion of the Se sublattice (∼6%) was formed by partially substituting surface Cu^+^ with Pb^2+^ [40]. The low diffusivity of Pb^2+^ ions into Cu_2_Se lattice and the absence of preferred entry positions in cubic Cu_2_Se led to the formation of a face-centered cubic Se anion sublattice, which was consistent with the host Cu_2_Se nanocrystals. During reaction, the moderate expansion of the Se sublattice was revealed to be critical in reducing strain and defects at the interface (Fig. 8f–h).

**Fig. 9:**
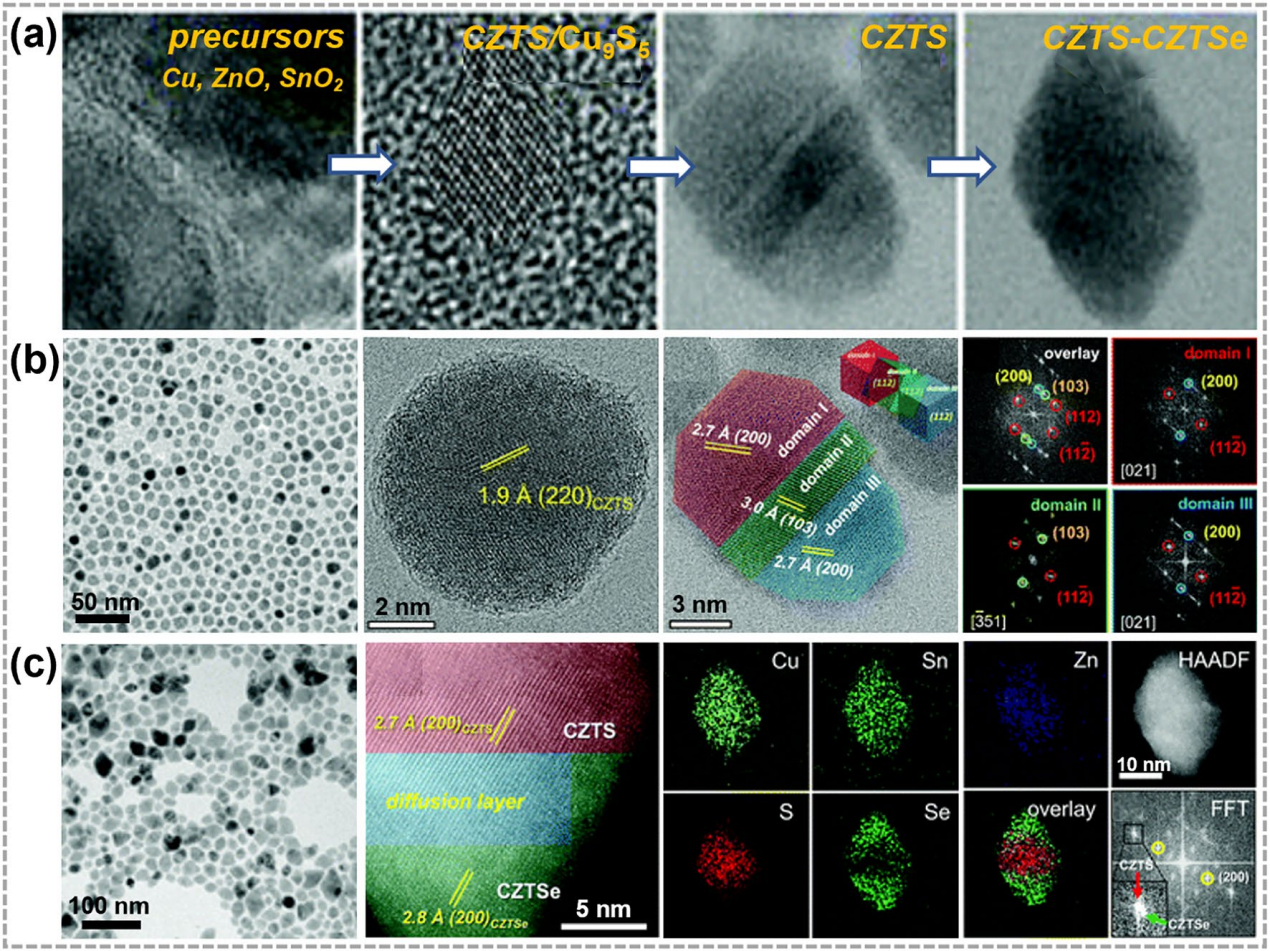
Synthesis and characterizations of 0D TMC heterostructures obtained by a combining CE and AE strategy. **a** TEM and HRTEM images of synthesis Cu_2_ZnSnS_4_-Cu_2_ZnSnSe_4_ (CZTS-CZTSe) nano-heterostructure process. **b** TEM images of CZTS nanocrystals and fast Fourier transform (FFT) patterns of overlay, domain I (red), domain II (green), and domain III (blue). **c** TEM and high-resolution scanning TEM (HR-STEM) images of CZTS-CZTSe heterostructures and corresponding STEM-EDS elemental mapping images as well as corresponding FFT pattern of CZTS-CZTSe nanocrystals. Reproduced with permission [128]. Copyright 2021, RSC Pub. (Color figure online)

Moreover, CE and AE can be combined to synthesize high-quality heterostructures. Yin et al. reported the synthesis of quaternary kesterite Cu_2_ZnSnS_4_-Cu_2_ZnSnSe_4_ (CZTS-CZTSe) heterostructures by a combining CE and AE strategy, in which Cu, ZnO and SnO_2_ were chosen as precursors (Fig. 9a). The small lattice mismatch between Cu_9_S_5_ and CZTS nanocrystals allowed a transformation from Cu_9_S_5_ to CZTS with minimal changes in the S^2−^ framework. This not only facilitated the incorporation of Zn and Sn but also helped to preserve the morphology of Cu_9_S_5_ nanocrystal (Fig. 9b). The pristine CZTS nanocrystals then served as a template for subsequent AE. HRTEM images in Fig. 9c show that Se treatment triggered the substitution of S^2−^ within kesterite CZTS surface by Se^2−^ during the AE process, and sandwich CZTS-CZTSe nano-heterostructures were obtained [128]. Compared with direct synthesis of pure kesterite CZTSSe nanoparticles, sandwich CZTS-CZTSe nano-heterostructures containing quaternary and quinary phases are more promising in promoting electron–hole separation in solar cell devices. Meanwhile, AE reactions overcame the energy barrier and energy difference between polycrystalline states that cannot be ignored in direct synthesis of CZTSSe nanocrystals.

In summary, the ion exchange in 0D TMCs is a reversible reaction, in which both the kinetic control and thermodynamic control play essential roles in regulating the overall process. By the complete cation substitution, the synthesized core@shell heterostructure with substantial lattice mismatches exhibits atomically organized interface. The independence of shell from the core is beneficial for overcoming the strain at the interface imposed by epitaxial strategy. During the anion exchange, the adding of extra reagents provides new possibilities for the synthesis of desired nanocrystals. In partial ion exchange, the realization of controllability is a crucial point in achieving TMCs with exceptional properties. Numerous studies have demonstrated that by preciously controlling the reaction time and reactant concentration, it is possible to obtain heterostructures and alloys with desired compositions and morphologies. These studies also pave the way for controllable doping.

## 1D TMCs

Due to their adjustable quantum confinement effects, 1D TMC nanostructures hold great potential in applications of photonics and thermoelectricity [28, 129]. Compared with the conventional vapor–liquid-solid methods, atomic substitution offers an alternative route to create 1D TMC materials with intricate structures and notable compositional diversity [39, 101]. Similar to the case of 0D materials, low-cost colloidal methods are commonly employed for synthesizing 1D TMC materials. In this section, the development of ion exchange reaction in preparing nanorods, nanowires and nanotubes, is summarized.

### Synthesis of 1D Nanorods

1D nanorods refer to nanostructures with one dimension much larger than the other two, exhibiting a distinct rod-like shape. This specific geometry enables the fine-tuning of their properties by varying the aspect ratios. To explore their fundamental properties and potential applications, great efforts have been dedicated to manipulate the morphologies and compositions of nanorods [28, 129–132].

**Fig. 10:**
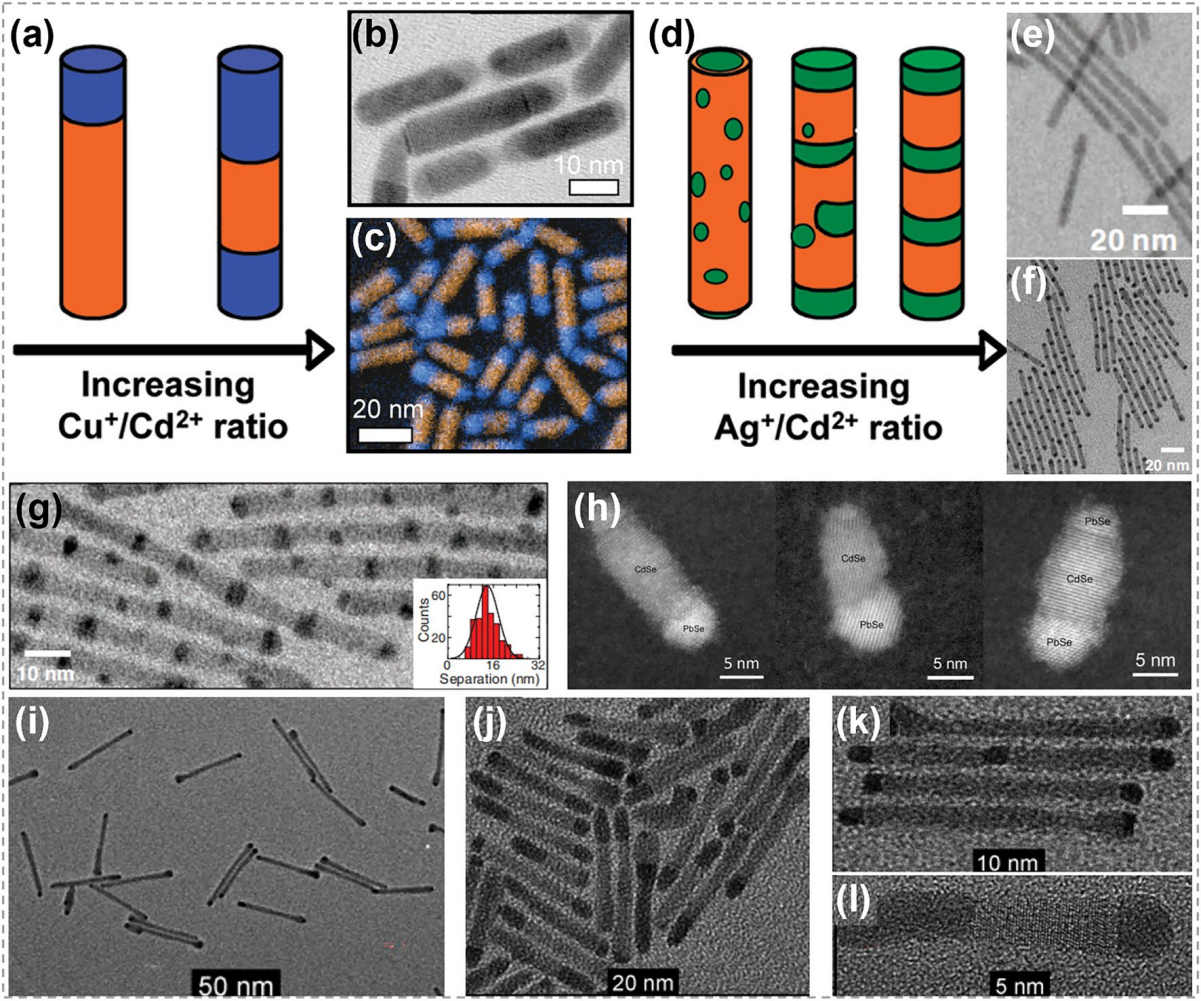
Synthesis of hetero-nanorods by ion exchange process. **a** The morphology of CdS-Cu_2_S nanorods by CE reactions. **b, c** TEM image and color-composite energy-filtered transmission electron microscopy (EFTEM) image of the obtained CdS-Cu_2_S nanorods. **d** Illustration of CdS-Ag_2_S nanorods produced by CE methods. Reproduced with permission [133]. Copyright 2009, American Chemical of Society. **e, f** TEM images of the pristine CdS nanorods and obtained CdS-Ag_2_S nanorods. **g** TEM images of CdS-Ag_2_S nanorods with inset showing histogram of Ag_2_S segment spacing (center-to-center). Reproduced with permission [131]. Copyright 2007, The American Association for the Advancement of Science. **h** HAADF-STEM image of CdS/Pd_4_S hybrid nanorods. Reproduced with permission [134]. Copyright 2015, American Chemical Society. **i** Tip growth of Pd_4_S on CdS nanorods with a diameter of 5.9 nm. **j** The extensive growth of Pd_4_S on CdS nanorods with a broad size distribution. **k** Close-up of the sample shown in **i**. **l** HRTEM of a single CdS-Pd_4_S nanorod from the sample shown in **j**. Reproduced with permission [11]. Copyright 2011, John Wiley and Sons

The substitution between atoms in nanorods is demonstrated to be selective, which plays an important role in determining the structure of products. For example, Sadtler et al. studied the partial transformation of CdS nanorods through CE reactions, as shown in Fig. 10a–g [131, 133]. In the case of Cu^+^–Cd^2+^exchange, the reaction started preferentially from either one or both ends of CdS nanorod, depending on Cu^+^/Cd^2+^ratio. This led to the growth of one or two Cu_2_S domains inward from the tip region. As a result, segmented Cu_2_S/CdS heterostructures were formed, in which the two subunits shared a flat interface perpendicular to the axial direction (Fig. 10a–c). The reason behind the selective nucleation and growth of Cu_2_S within CdS nanorods is the exceptional stability of formed CdS-Cu_2_S interfaces. In the case of Ag^+^-Cd^2+^exchange, the reaction occurred non-selectively and the formed Ag_2_S regions are randomly distributed within CdS nanorod. During the CE process, multiple Ag_2_S segments spanned the diameter of nanorod with a uniform size (Fig. 10d–g). The non-selective nucleation of Ag_2_S in CdS nanorod was ascribed to the negative chemical formation energies of CdS-Ag_2_S interface. However, as Ag_2_S regions extended within the nanorods, the influence of elastic energy became dominant, driving the ripening process of Ag_2_S regions and resulting in the reduction of interfacial area. The randomly distributed Ag_2_S regions finally evolved into a periodic pattern. The distinctions between CdS–Cu_2_S and CdS–Ag_2_S systems arise from differences in both the chemical favorability of bond formation and the elastic distortions at the interfaces. This offers an opportunity to manipulate the spatial configuration of components within heterostructures, and further control their chemical and physical properties.

The CdX/PbX system (*X* = S, Se, Te) has also been widely studied due to their excellent photoelectric property and chemical stability. Because PbX and CdX are immiscible, the exchange between Cd^2+^ and Pb^2+^ generally results in separated CdX/PbX heterostructures. Lee et al. demonstrated that the exposure of CdSe to Pb^2+^ ions lead to the transformation of wurtzite CdSe into rock-salt PbSe [134]. As shown in Fig. 10h, the replacement of Cd^2+^ ion was found to occur anisotropically, starting from the tips of nanorods and leading to the formation of interfaces parallel to the (0001) plane of nanorods. This is similar to the CdS/Cu_2_S case depicted in Fig. 10b [133]. Moreover, the Cd-to-Pb exchange was facet-selective and two tips of nanorods showed significant differences during transformation, because the (000 $\overline{1}$) facet of CdSe nanorods is more active than the (0001) one. Explorations in this area enable precise control at the atomic level for CE, which could expand the possibility for designing CdX/PbX heterostructures. Under the similar reaction conditions, Zhang et al. prepared CdS/PbS and CdSe/PbSe Janus-like heterostructures by partial CE reactions [135]. The obtained structure exhibited excellent optoelectronic properties that can be customized for potential applications of various fields. By exposing CdS nanorods to Pd^2+^ containing solution, Shemesh et al. realized the synthesis of CdS/Pd_4_S segmented nanorods [11]. Similarly, the formation of Pd_4_S started from either one or both tips of CdS nanorods, resulting in the synthesis of segmented CdS/Pd_4_S structures with planar interfaces (Fig. 10i–l). These planar interfaces are critical in reducing interface energy and strain, thereby ensuring the stability of the structure.

### Synthesis of 1D Nanowires

**Fig. 11:**
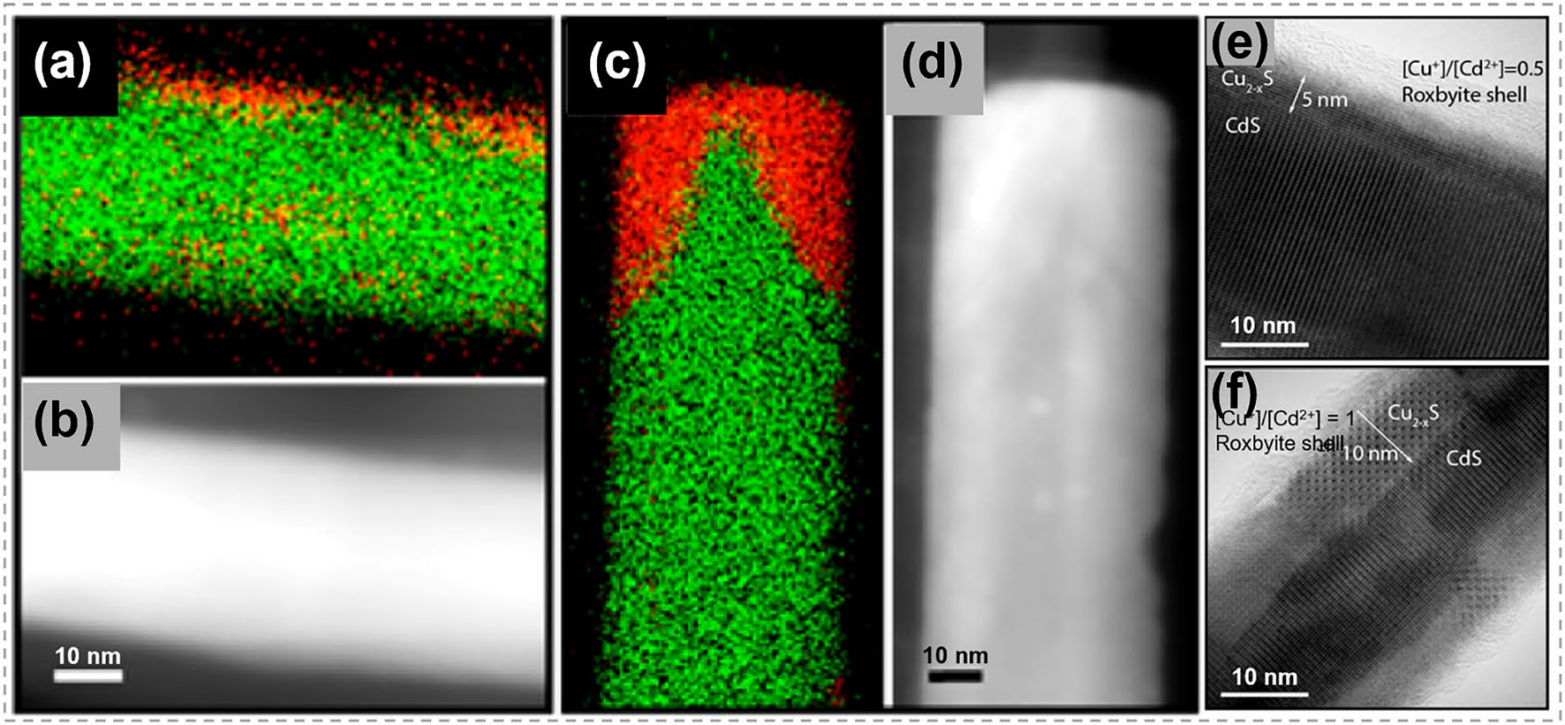
Synthesis and characterization of CdS@Cu_2-x_S nanowires by ion exchange process. **a-d** EDS element mapping images for Cd (in green) and Cu (in red) and corresponding STEM images. **a, b** Images showing the substitution started at the side surface of nanowires. **c, d** Images showing the substitution started at the tips of nanowires. **e, f** HRTEM images of CdS@Cu_2–x_S core@shell nanowires. **e** A core@shell nanowire obtained with a 0.5:1 Cu^+^/Cd^2+^ ratio, **f** a core@shell nanowire with increasing shell thickness obtained with a 1:1 Cu^+^/Cd^2+^ ratio. Reproduced with permission [129]. Copyright 2014, American Chemical Society. (Color figure online)

By CE reactions, Zhang et al. synthesized CdS@Cu_2_S core@shell nanowires with diameters of 30–40 nm and lengths of ~ 10 μm, as exhibited in Fig. 11 [129]. It was found that Cu^+^–Cd^2+^ exchange reaction occurred simultaneously at both side facets (Fig. 11a, b) and tips (Fig. 11c, d) of CdS nanowires, with a notable preference at the tips. The calculated transformation energy for nucleation from side facets was approximately seven times higher than that at tips [133]. Furthermore, solid-state diffusion enabled the penetration Cu ions into the inner regions of the CdS nanowires. This diffusion process resulted in the formation of core@shell nanowires, and the thickness of the shell increased as the reaction continued. Besides, the composition of resulting core@shell nanowire was found to be dependent on the Cu^+^/Cd^2+^ ratio. As shown in Fig. 11e, f, CdS@Cu_2-x_S nanowires were achieved under a Cu^+^/Cd^2+^ ratio of 0.5:1, while CdS@Cu_2_S nanowires with a thicker shell were formed under a Cu^+^/Cd^2+^ ratio of 1:1. Similar with the ion exchange reaction in 0D TMC alloys (Fig. 6d–f), by regulating the reactant concentration, this research provides a method for precisely control over the structure, composition, and crystal phases of nanowires, holding a promising prospect in various applications.

**Fig. 12:**
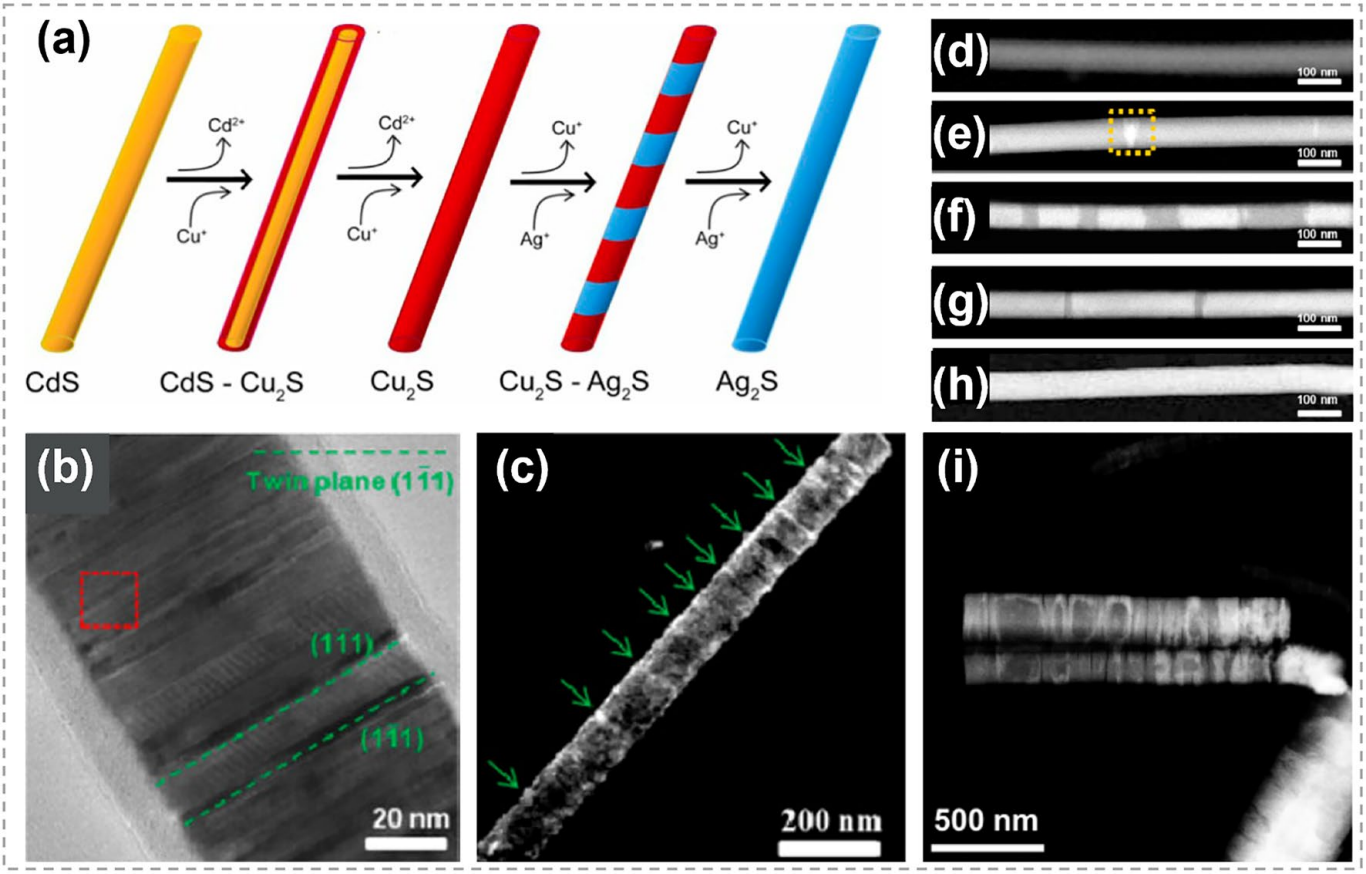
Synthesis and characterizations of Cu_2_S-Ag_2_S heterostructure nanowires. **a** Illustration shows the formation process of Cu_2_S-Ag_2_S superlattice nanowires. **b** TEM image of a Cu_2_S nanowire transformed from a CdS nanowire. The twin planes are the (11̅1) planes. **c** Dark-field TEM image of a single Cu_2_S nanowire viewed along the [109] zone axis and presented by selecting the (11̅1) diffracted beam. The green arrows indicate locations of the (11̅1) twins. **d-h** HAADF-STEM images of the Cu_2_S-Ag_2_S nanowires formed. Reaction times are **d** 0 s, **e** 6 s, **f** 24 s, **g** 48 s, **h** 60 s. **i** HAADF-STEM image of Ag_2_S/Cu_2_S nanowires after CE reactions in monoclinic Cu_2_S structure. Reproduced with permission [28]. Copyright 2014, American Chemical Society. (Color figure online)

Tan et al*.* realized the controllable synthesis of multiple p-n segmented heterojunctions by a two-step CE strategy, as illustrated in Fig. 12a. Using single-crystal CdS nanowires as reactants, Cu_2_S nanowires with face-centered cubic (*fcc*) crystal structure were obtained after the complete exchange of Cd^+^ by Cu^+^. Figure 12b, c shows that there are abundant twin planes perpendicular to axial direction [28]. These twin boundaries offered active sites for the following exchanging between Cu^+^ and Ag^+^. The newly formed Ag_2_S segments were found to be parallel to the twin planes and elongate along the axial direction with the reaction proceeded, as shown in Fig. 12d–h. This suggests that the morphology of regular Ag_2_S–Cu_2_S heterojunctions can be well controlled by terminating CE process at a precise reaction time. This work highlights the significance of crystal structure and orientation of twin plane in determining the regularity and pattern of the resulting Cu_2_S–Ag_2_S heterostructures. As a comparison, irregular patterning was observed after Cu^+^–Ag^+^ exchange reaction in monoclinic Cu_2_S nanowires with twin planes not perpendicular to axial direction (Fig. 12i). These explorations provide two effective methods to control the morphology of nanowires by regulating the reactant concentration and reaction time, respectively. In addition, the ion exchange rate demonstrates noticeable location selectivity in both of the above studies.

### Synthesis of 1D Nanotubes

**Fig. 13:**
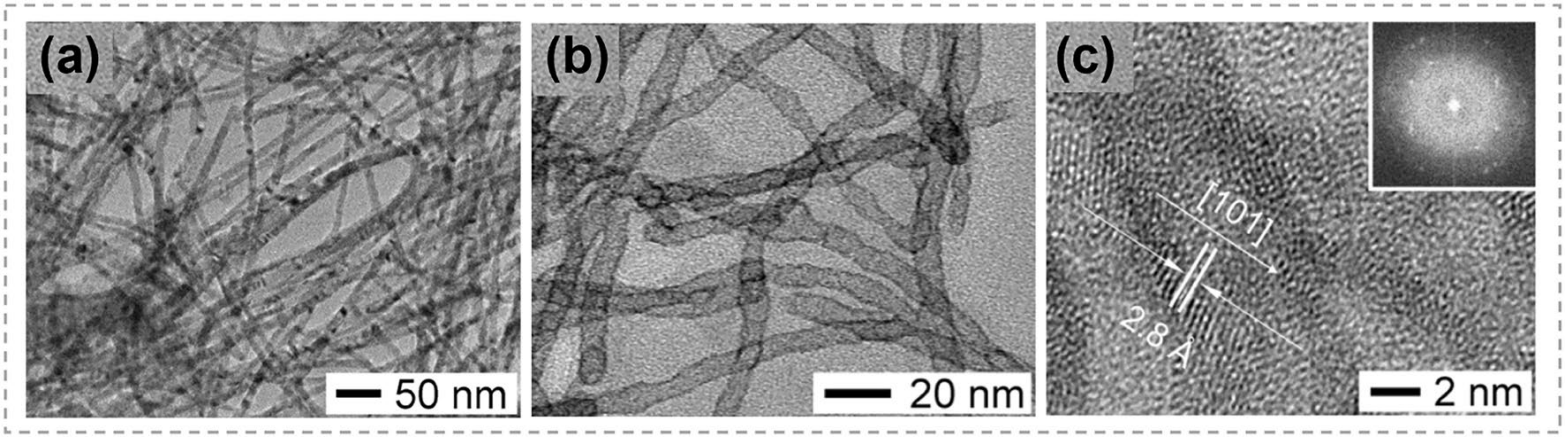
Synthesis of PtTe_2_ nanotubes via the shape evolution in Cd^+^-Pt^4+^ exchanging process. **a** TEM image of CdTe nanowires, **b** TEM image of PtTe_2_ nanotubes derived from CdTe nanowires through CE reactions. **c** HRTEM image of a PtTe_2_ nanotube indexed as the hexagonal phase with lattice spacing of 0.28 nm along the [100] direction. The inset shows the Fourier transformed ring pattern of PtTe_2_ nanotubes. Reproduced with permission [39]. Copyright 2010, American Chemical Society

**Fig. 14:**
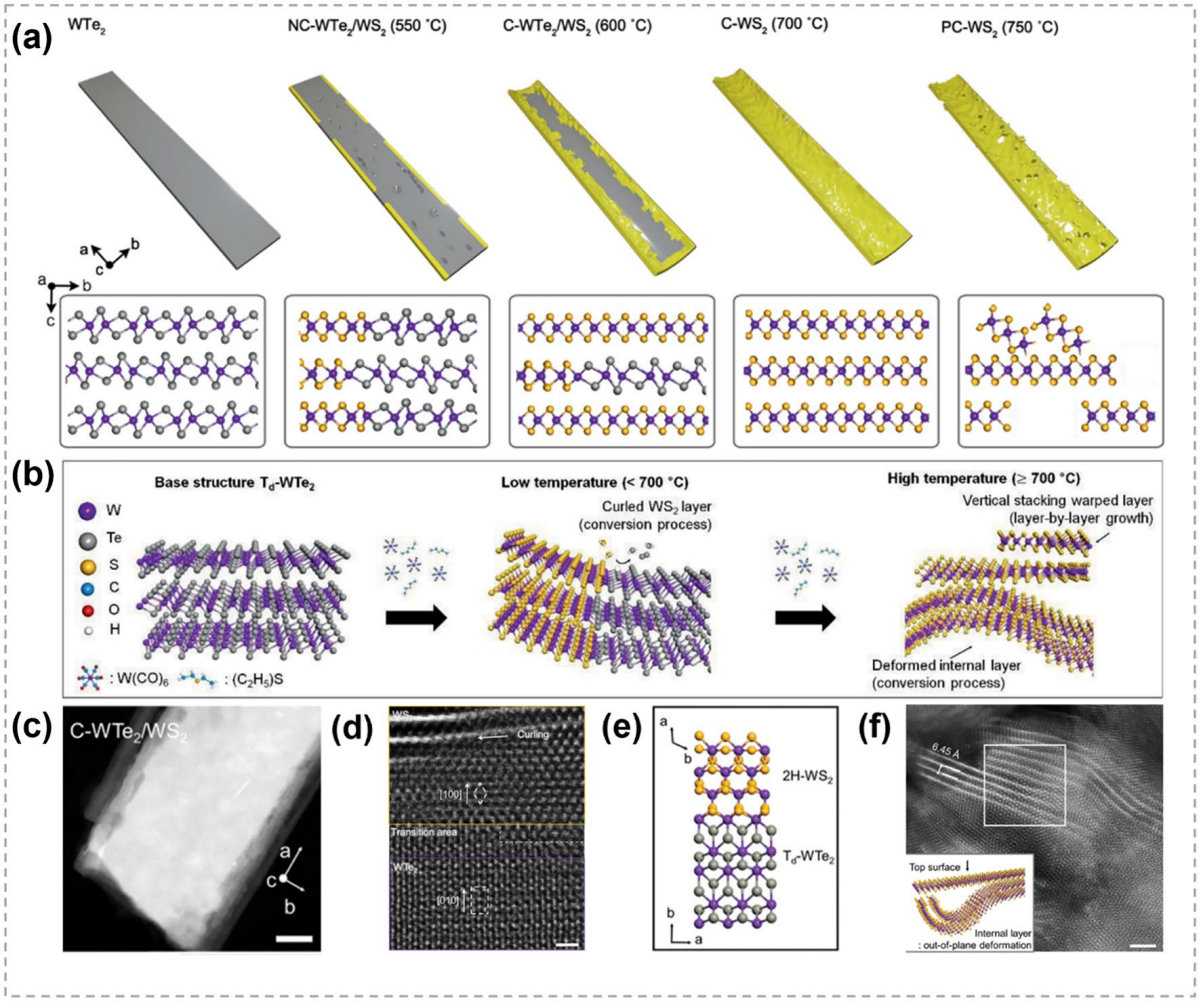
Structure diagram and characterizations of TMC nanobelts by atomic substitution process. **a** The temperature-dependent synthesis of nanobelts via the substitution of chalcogen. NC-WTe_2_/WS_2_ represents non-curled WTe_2_/WS_2_, C-WTe_2_/WS_2_ represents curled WTe_2_/WS_2_ and C-WS_2_ represents curled WS_2_. **b** Schematic showing controlled conversion process at different temperatures. **c** HAADF-STEM image (scale bar: 30 nm) of the curled WTe_2_/WS_2_ grown at 600 °C. **d, e** Atomic-Resolution STEM image (scale bar: 1 nm) and atomic configurations showing the interface structure of the curled WTe_2_/WS_2_ heterostructure. **f** Plan-view Atomic-Resolution STEM image (scale bar: 2 nm) and atomic model showing the WS_2_ layer was deformed. Reproduced with permission [136]. Copyright 2020, John Wiley and Sons

By taking advantage of large volume change during a certain CE reaction, Moon et al*.* realized the transformation of nanowires to nanotubes, as shown in Fig. 14 [39]. Ultra-thin Ag_2_Te nanowires were chosen as reactants, and were converted into CdTe nanowires in a Cd^+^ containing solution. During this process, the single crystalline structure of nanowires was preserved and the volume change was slight (Fig. 13a). Afterward, these CdTe nanowires were exposed to Pt^4+^ ions, and PtTe_2_ nanotubes with a wall thickness of 1–2 nm were formed. During this process, the mechanical stress caused by CE accumulated continuously, inducing the creation of voids in products. As a result, the single crystalline nanowires were transformed into polycrystalline PtTe_2_ nanotubes (Fig. 13b, c). This research illustrates the important effect of stress in the morphology transformation and is valuable for understanding the shape transformations in nanostructured materials under large stress.

### Synthesis of 1D Nanobelts

In 2020, Sim et al*.* introduced a novel approach for designing TMC heterojunctions by Metal Organic CVD [136]. Single-crystalline WTe_2_ nanobelts were adopted as the base structures and then sulfurized, as schematically shown in Fig. 14a. Depending on sulfurization temperature, Te atoms were replaced by S to different extents and various structures were formed, including non-curled WTe_2_/WS_2_, curled WTe_2_/WS_2_, curled WS_2_, and porous/curled WS_2_ as illustrated in Fig. 14b. At low temperatures, the substitution of Te by S predominantly started from edges, and the single-crystalline structure was maintained. At high temperatures, the conversion resulted in structurally deformed WS_2_ throughout the entire layers of nanobelt. Figure 14c shows the obtained curled WTe_2_/WS_2_ at 600 °C. The presence of an atomically ordered interface in Fig. 14d, e indicates that the lattices of two subunits were maintained in a coherent manner across the transition region, suggesting the heteroepitaxial stacking of WTe_2_/WS_2_ hybrids despite of their large lattice mismatch (≈8.8%). Figure 14f presents the as-prepared curled WS_2_ under 700 °C, despite many pores and deformed layers were observed throughout the entire area, the shape of nanobelt remained intact. Remarkably, the obtained 1D heterojunctions showed enhanced performance in the hydrogen evolution reaction as well as the long-term durability for electrocatalytic reactions, owing to the increased effective surface area and reduced electron-transfer resistance. Though the specific mechanism is not clear, the temperature effect in this research provides great reference for the exploration of the transformation between 1D TMCs.

Similar to the cases of 0D TMCs, the ion exchange method in 1D TMCs have illustrated the effects of temperature, reactant concentration and reaction time on both the composition and morphology, providing valuable references for controllable synthesis. Additionally, strain, surface energy and interfacial energy also show noticeable influences on the structural evolution during the substitution process. Moreover, both the exchange rate and initiation sites exhibit significant location dependence during the reaction.

## 2D TMCs

2D TMCs predominantly have a chemical formula of MX_2_, where M represents transition metals from Group IVB to Group VIIIB. In a 2D TMC structure, atoms within the same layer are chemically bonded, while those between layers interact through weak van der Waals force [9, 137, 138]. Depending on their compositions and phase structures, 2D TMCs display various electronic properties [139–145], showing great potential in applications of electronic devices, quantum devices, energy catalysis [146–149], etc.

In recent years, atomic substitution has been widely used in regulating the compositions and structures of 2D TMCs, and various materials have been obtained, including binary compounds, doped materials, alloys and heterojunctions [20–23, 150, 151], etc. Unlike the solution methods that are widely employed in 0D and 1D TMCs, CVD techniques are very popular for implementing atomic substitution process in 2D TMCs.

### Initiation of Atomic Substitution

Owing to the ultra-thin structure, 2D TMCs provide a unique platform for studying the real-time process of atomic substitution. It is revealed that crystal defects, such as vacancies, grain boundaries (GBs), surfaces and edges, serve as starting points for the substitution process.

#### Vacancy-Initiated Substitution

**Fig. 15:**
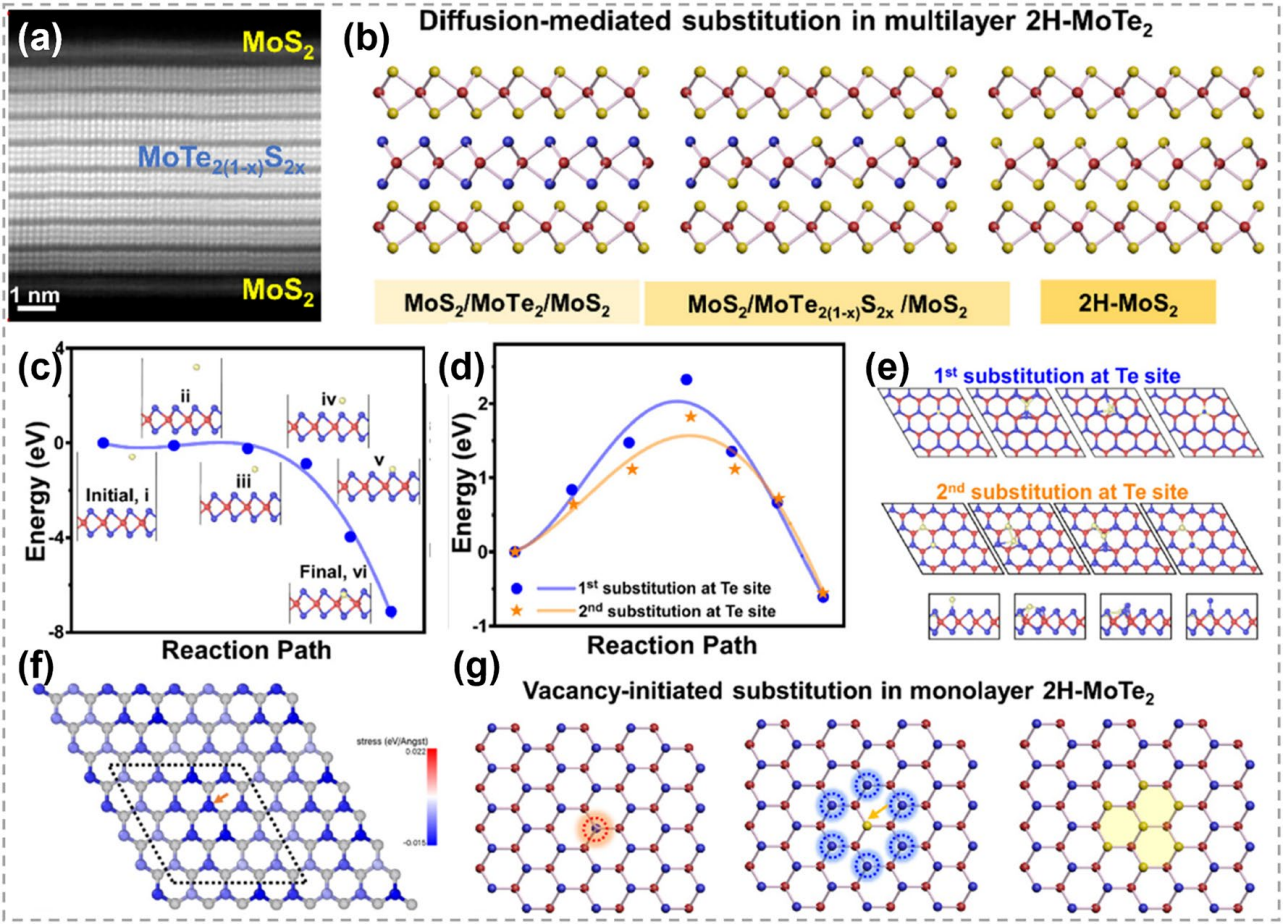
Mechanism of atomic substitution initiated by vacancies. **a** Magnification HAADF-STEM cross-sectional image of the sulfurized MoTe_2_. **b** Diffusion-mediated substitution mechanism in multilayer MoTe_2_ (Red: Mo, yellow: S, and blue: Te) **c** Energy barrier for S substitution at Te vacancy. Insets are the path details. **d** Energy barriers and **e** path details for S substitution at Te sites (blue) in the perfect MoTe_2_ (orange) and after the insertion of first S. **f** Force map after the replacement of first S. **g** Diffusion-mediated substitution mechanism in multilayer MoTe_2._ Reproduced with permission [152]. Copyright 2021, American Chemical Society. (Color figure online)

By sulfurizing sub-centimeter scale single crystalline 2H-MoTe_2_ thin film, Liu et al. achieved a corresponding single crystalline MoS_2_/MoTe_2(1-x)_S_2x_/MoS_2_ sandwich structure and proposed a Te vacancy-initiated and S diffusion-mediated transformation mechanism (Fig. 15a, b) [152]. The S substitution process started at Te vacancies in the top and bottom layers, and then progressed into the middle layers. Though the rate of substitution in the middle layers is limited by cross-layer diffusion of S atoms, the complete transformation is possible with the continuation of the process. Density functional theory (DFT) calculations also confirmed the role of Te vacancy, and found that there is no energy barrier for the occupation of S at Te vacancy site (Fig. 15c). In comparison, the energy barriers for the 1st and 2nd substitutions of Te in perfect MoTe_2_ structure are ~ 2.3 and 1.8 eV, respectively (Fig. 15d, e). It was noted that the substitution process is thermodynamically favorable. The lower energy barrier of the 2nd substitution was attributed to the strain field induced by the 1st substitution, as evidenced by the force mapping analysis (Fig. 15f). The compression caused by the initial substitution of S was beneficial for reducing the energy barrier of subsequent substitutions (Fig. 15g). This work provides an efficient way to acquire large-scale high-quality TMC films and heterostructures. Similarly, Taghinejad et al. realized the fabrication of MoS_2x_Se_2(1-x)_ alloy via sulfurizing MoSe_2_ films. It was revealed that the driving force of substitution is much lower in CVD-grown MoSe_2_ films with abundant vacancies than that in exfoliated films with fewer vacancies [153].

The present of vacancies also plays an important role in promoting the substitution between metal atoms. By combining DFT calculations and experiments, Liu et al. [154] explored the Co substitution in MoS_2_ and revealed that the substitution is induced by sulfur vacancies. Chang et al. [155] found that the metal exchange between Sn and W can be promoted by producing S vacancies at high temperatures. To date, many strategies have been developed for vacancies creation to promote substitution processes, such as high temperature, ion beam bombardment and gaseous plasmas [21, 155, 156].

#### Grain Boundary-Initiated Substitution

**Fig. 16:**
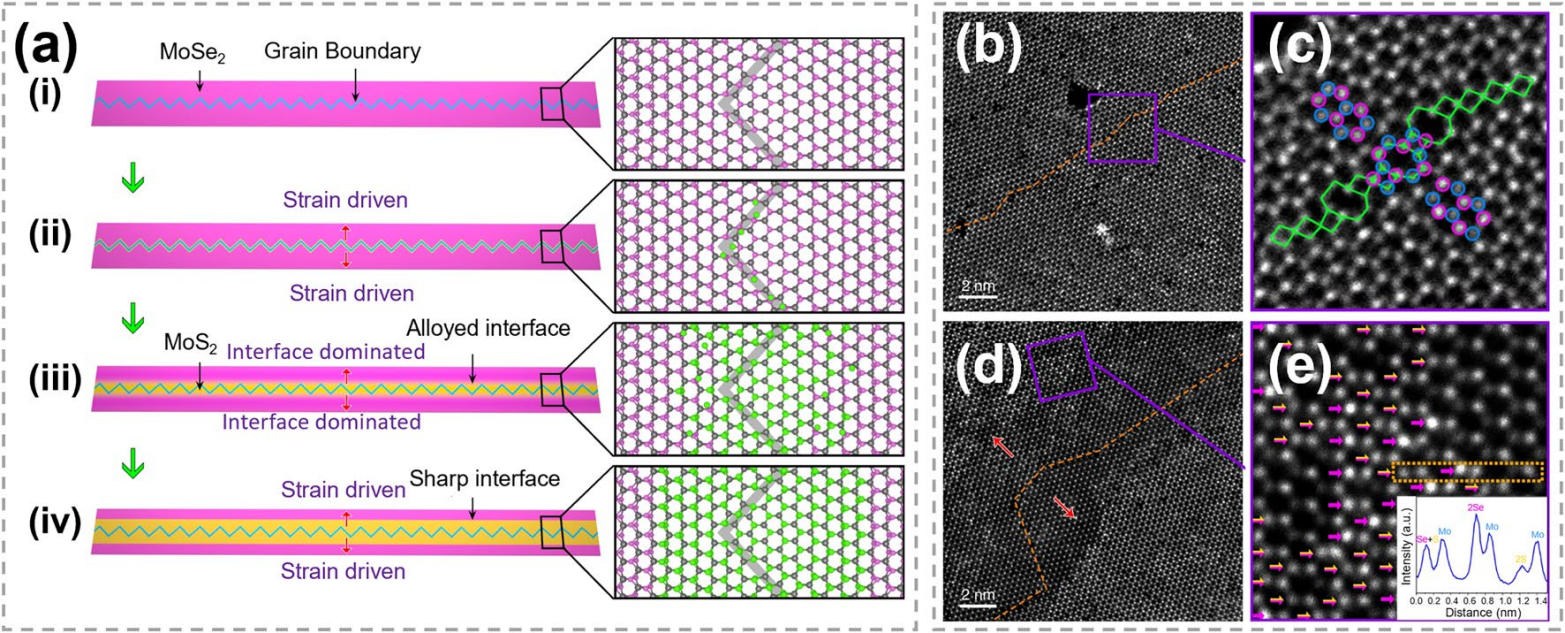
Mechanism exploration in atomic substitution initiated by grain boundary. **a** Schematic showing growth of MoS_2_ channels. **b, c** Annular dark-field scanning transmission electron microscopy (ADF-STEM) images of intrinsic 60° GB (orange dashed line) within pristine monolayer MoSe_2_ and atomic arrangement of this GB (blue and purple circles: Mo and Se atoms) resembles that in MoS_2_ nano-channels. **d, e** ADF-STEM images showing part of MoSe_2_-MoS_2_ hybrid winding channel. The red arrows represent the sulfidation direction. Purple arrows mark 2Se atoms and yellow-purple arrows mark S + Se atoms, with line intensity profile showing in the inset. Reproduced with permission [157]. Copyright 2020, Spring Nature. (Color figure online)

GBs have also been demonstrated to be initiation sites for atomic substitution. As shown in Fig. 16a, starting from intrinsic GBs of MoSe_2_, Zhu et al. obtained ultra-long MoS_2_ nano-channels successfully [157]. Figure 16b, c displays the pristine MoSe_2_ monolayer, where lots of active sites in 8|4|4|8 GBs can be observed. The formation process of MoS_2_ can be divided into three steps: (i) the chemisorption of S atoms near the GBs, (ii) the exchange between S and Se atoms, (iii) the desorption of Se atom. The second step has the highest energy barrier and is considered as the rate-limiting step. The energy barrier for replacing Se atoms at GBs is much lower, owing to the high in-plane tensile strain. The substituted S atoms then act as the nucleation center, leading to the continued growth of MoS_2_. Near the GB, MoSe_2_ was completely transformed into MoS_2_, while mixed structures that one Mo atom coordinates with S_2_, Se_2_, or S + Se atoms were formed at the interface of MoS_2_ and MoSe_2_, as shown in Fig. 16d, e. This study offers an excellent method for achieving narrow lateral heterostructures and precise control over the spatial scale of heterostructures.

#### Edge-Initiated Substitution

**Fig. 17:**
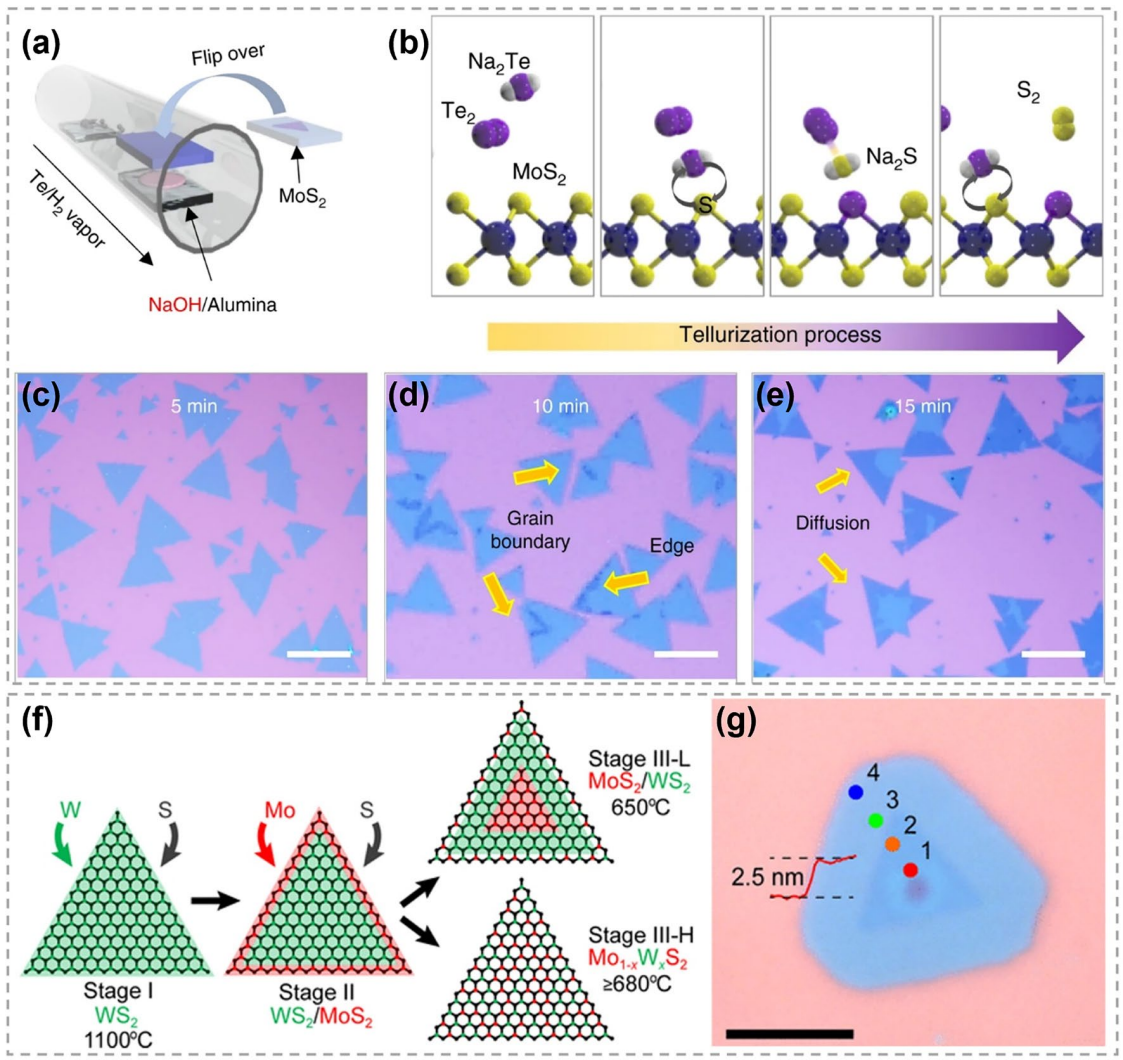
Mechanism of atomic substitution initiated by edge. **a** Alkali-metal-assisted transformation from MoS_2_ to MoTe_2_. **b** Schematic showing Na-assisted tellurization process. **c-e** Optical images showing the evolution from MoS_2_ to MoTe_2_. Reproduced with permission [158]. Copyright 2017, Springer Nature. **f** Illustration showing the substitution of W in WS_2_ by Mo at different temperatures (W atoms are shown in green, Mo atoms are red, S atoms are black). **g** Optical image of a typical heterostructure revealing a distinct core-ring structure. Scale bar is 5 μm. Reproduced with permission [159]. Copyright 2016, American Chemical Society. (Color figure online)

The enhanced reactivity of TMCs edges, attributed to their unsaturated chemical bonds, makes them more favorable for atomic substitution compared to the less reactive basal plane [136]. As displayed in Fig. 17a–e, Yun et al. converted monolayer MoS_2_ to MoTe_2_ in a Te-rich environment with the assistant of NaOH [158]. During the substitution process, Na_2_Te, which is the most probable Na-Te compound, acted as the driving agent to accelerate the tellurization of MoS_2_ and the dissipation of gaseous S_2_ (Fig. 17b). The optical images in Fig. 17c–e show the tellurization progress of MoS_2_. It is clear that MoTe_2_ primarily emerged along the edges and GBs, gradually extending toward the central area of MoS_2_. In contrast, the occurrence of substitution was not limited at edges at high temperatures, leading to random tellurization over MoS_2_ basal plane. Similarly, Bogaert et al. investigated the effect of temperature on the substitution between W and Mo in as-grown WS_2_ islands by CVD methods (Fig. 17f) [159]. At a relatively low temperature (650 °C), Mo substitution predominantly took place at WS_2_ edges. As a result, an in-plane MoS_2_/WS_2_ heterostructure with a sharp interface was formed (Fig. 17g). At a relatively high temperature (710 or 680 °C), the entropy contribution to the Gibbs free energy increased and led to the formation of Mo_1-x_W_x_S_2_ alloy. Therefore, it is possible to customize the pathway to form either TMC alloys or lateral heterostructures by controlling the substitution kinetics.

### Synthesis of 2D TMC Binary Compounds

#### Substitution Between TMCs

**Fig. 18:**
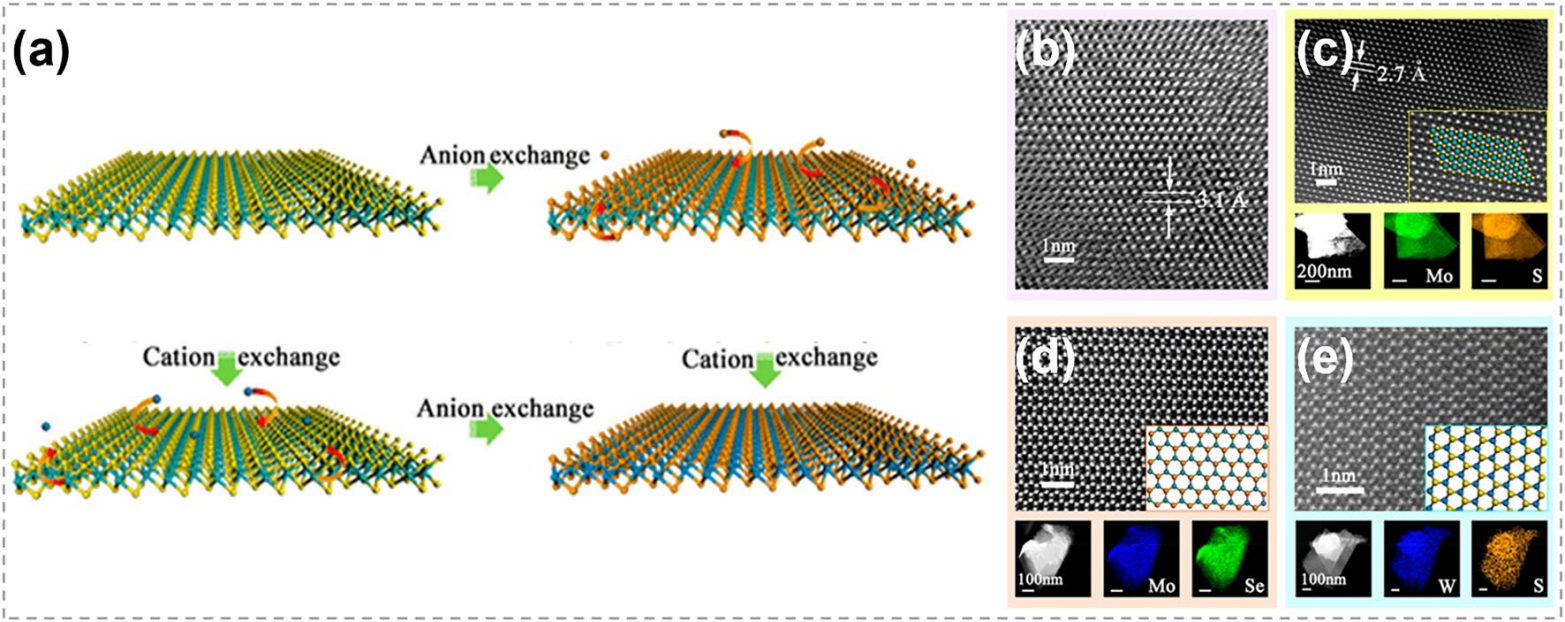
Transformation between different TMCs by atomic substitution. **a** Schematic showing ion exchange in TMCs. **b** HRTEM image of a SnS_2_ nanosheet before CE reactions. **c** HRTEM and element mapping images of a MoS_2_ after CE. **d** STEM and element mapping images of a MoSe_2_ after AE. **e** STEM and element mapping images of a WS_2_ after CE. Reproduced with permission [27]. Copyright 2017, American Chemical Society

The complete substitution of either transition metal atoms or chalcogen atoms within a TMC shows a great potential in converting the TMC to another type, as illustrated in Fig. 18a. By using exfoliated 2D TMC nanosheets as reactants, Duan et al. explored the feasibility and universality of ion exchange reactions in solution phase [27]. Three reactions were conducted successfully, including converting SnS_2_ into MoS_2_, converting MoS_2_ into MoSe_2_, and converting MoS_2_ into WS_2._ It was revealed that the ion exchange reactions were completely finished without specific adjustment of ion solubility, because the crystal structures and thermodynamic stabilities of the reactants and products were similar. HRTEM images in Fig. 18b–e show that the TMCs obtained by both CE and AE were highly crystalline with a relatively uniform elemental distribution. Compared with traditional CVD method, this work shows atomic substitution strategy as an alternative and flexible way to achieve new TMC materials.

#### Oxygen-Chalcogen Substitution

**Fig. 19:**
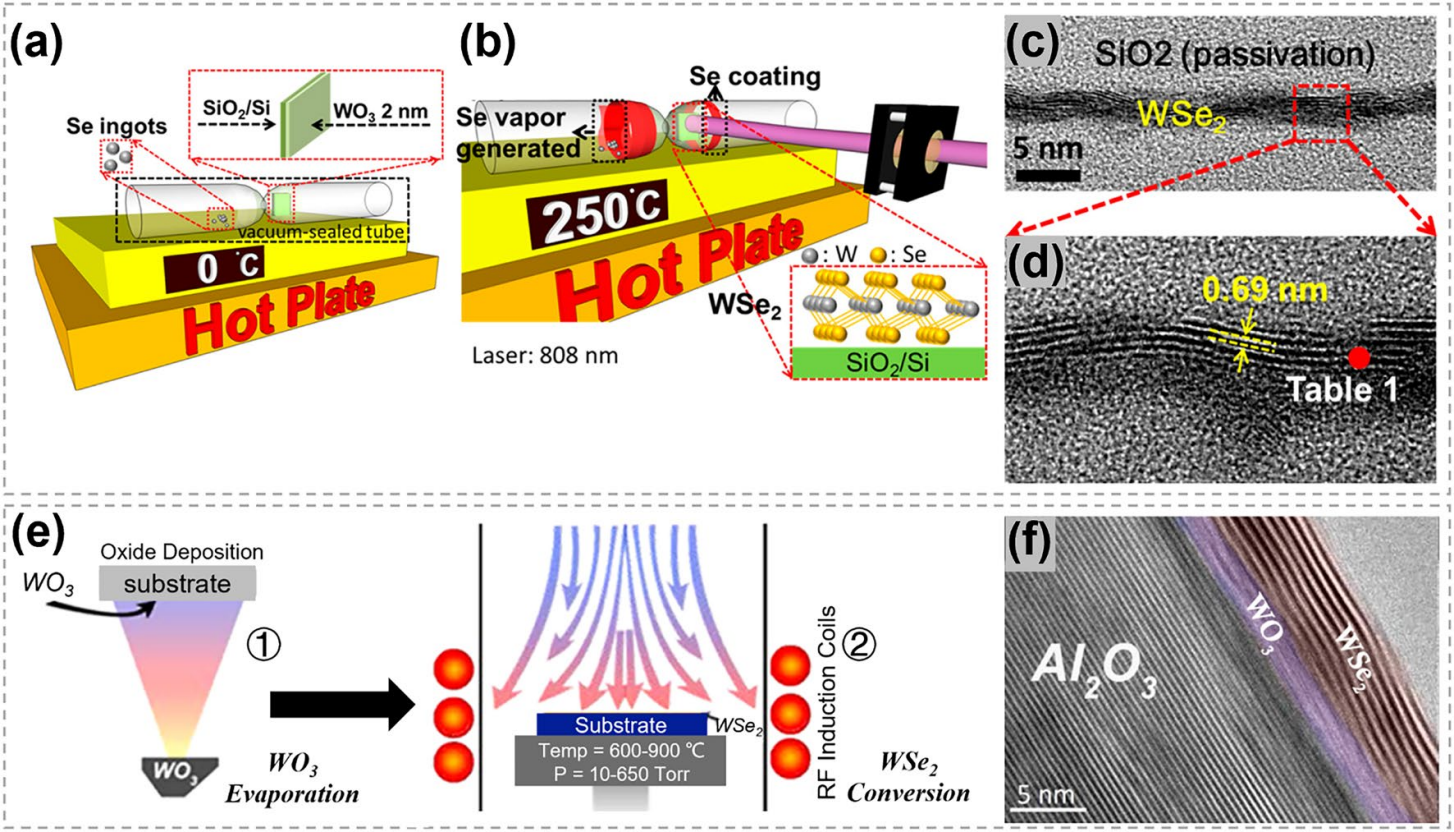
Synthesis of WSe_2_ by the oxygen-chalcogen exchange process. **a** Illustration showing 2 nm-thick WO_3_ on SiO_2_/Si and Se ingots was vacuum-sealed together. **b** Illustration showing the formation of WSe_2_ when heating up to 250 °C. **c** Across-sectional TEM image of WSe_2_. **d** HMTEM image revealing the interlayer distance of the WSe_2_. Reproduced with permission [160]. Copyright 2015, American Chemical Society. **e** Synthesis process of WSe_2_ converted from amorphous WO_3_ and hexagonal stabilized WO_3_ films. Thermal evaporated WO_3_ films were annealed via rapid thermal annealing and then converted to WSe_2_ via cold wall furnace. **f** TEM image of the obtained layered WSe_2_ structure and the unconverted WO_3_ film between sapphire and WSe_2_. Reproduced with permission [164]. Copyright 2015, IOP Publishing

It is very common to prepare TMCs through utilizing chalcogens to reduce transition metal oxides in traditional CVD methods. In the process, transition metal oxides powder usually go through a high-temperature melting phase to yield metal precursors, followed by the nucleation and growth of TMCs. In contrast, the oxygen-chalcogen substitution strategy, based on transition metal oxide films, allows for the avoidance of the melting process. Chen et al. reported the selenization of WO_3_ thin films to produce WSe_2_ with the assistance of laser [160]. As shown in Fig. 19a, b, a WO_3_ film was directly deposited on a SiO_2_/Si substrate and placed in a vacuum-sealed quartz tube with Se ingots. The quartz tube was then placed on a hot plate to generate homogeneous Se vapor and a continuous wave laser was utilized to trigger the substrate heating. In the presence of selenium gaseous vapor, the amorphous WO_3_ film underwent a reduction process when the laser was irradiating on it. Layered WSe_2_ film was successfully achieved, as shown in Fig. 19c, d. Besides, through a process involving the patterning of the WO_3_ film and subsequent laser irradiation, patternable WSe_2_ fabrication was realized. Compared with thermal CVD method, the laser assistant substitution can achieve an ultrafast heating on the target part, making the synthesis process to be more controllable. This laser assisted reduction process was also demonstrated to have a great potential in the synthesis of other TMCs, such as MoS_2_, WS_2_, WSe_2_ [161–163]. Large area WSe_2_ have been synthesized by direct selenization of as-deposited WO_3_ films [164]. As shown in Fig. 19e, the WO_3_ films were deposited onto substrates via thermal evaporation first and then converted to WSe_2_ by exposing to dimethyl selenium in a cold wall reactor. As shown in the cross-section TEM image (Fig. 19f), 8–10 layers of WSe_2_ were generated on the WO_3_ film. Besides, the thickness of WSe_2_ can be effectively controlled by the thickness of WO_3_ film and the depth of selenization. This research proposed an effective route to synthesize TMC films with controllable and highly uniform thickness based on the transition metal oxide.

#### Iodine-Chalcogen Substitution

**Fig. 20:**
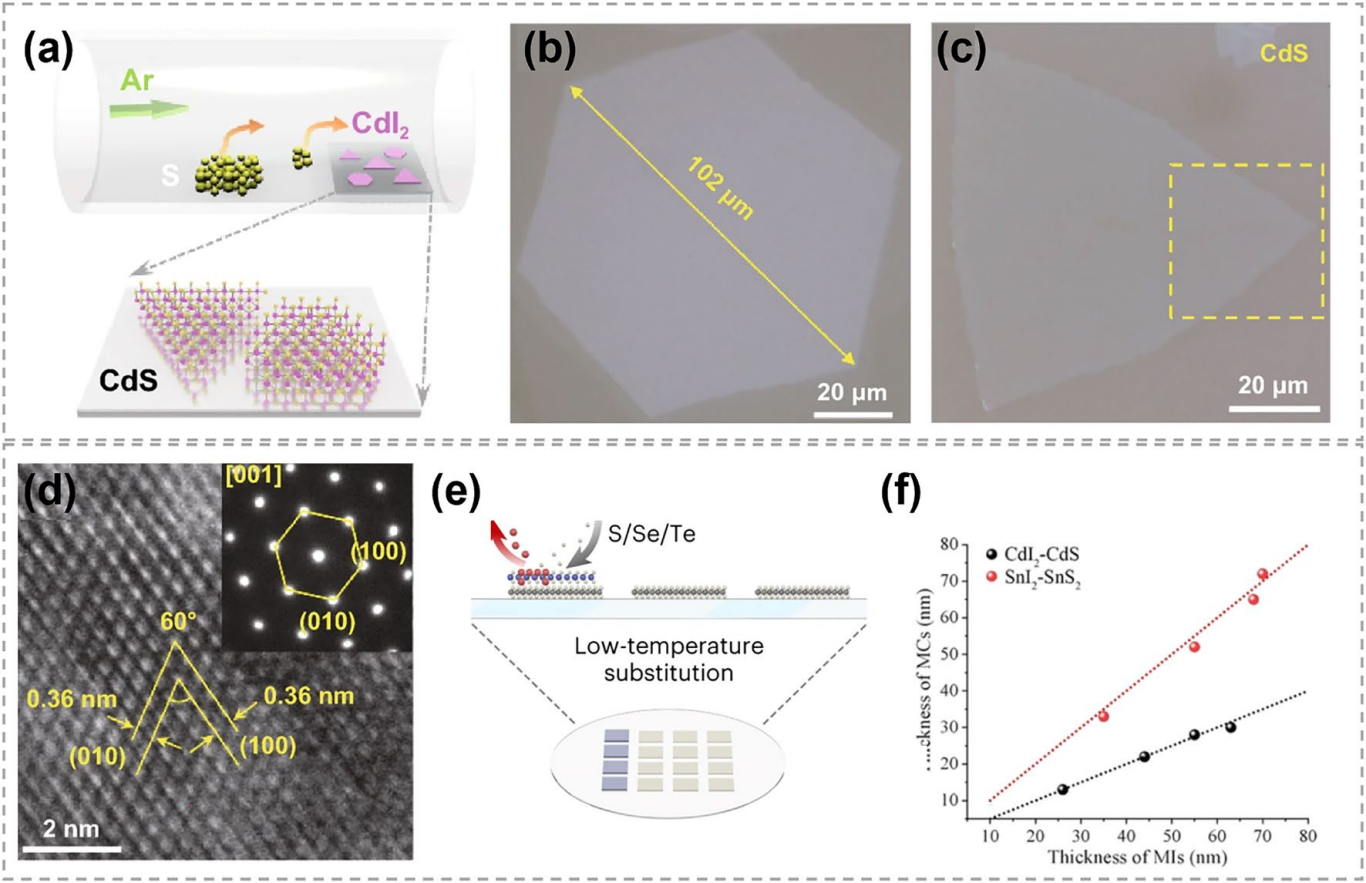
Chalcogen substituting iodine in the synthesis of TMCs. **a** Schematic of the substitution and atomic structure of CdS flakes on mica substrate by chemical sulfurization method. **b** Representative optical microscope images of converted CdI_2_ thin flake on mica substrates. **c** Representative optical images of original large-scale CdI_2_ thin flake and converted CdS thin flake on mica substrates, respectively. **d** HRTEM image of converted CdS flakes. The inset is the selected area electron diffraction pattern taken along the direction [001] of CdS flakes. Reproduced with permission [35]. Copyright 2021, Springer Nature. **e** Schematic showing the preparation of CdS flakes on mica substrate by chemical sulfurization method. **f** The thickness of CdI_2_ and SnI_2_ flakes after being converted to CdS and SnS_2_. Reproduced with permission [34]. Copyright 2023, Springer Nature

Owing to the low-energy-barrier substitution of iodine with other chalcogens, metal iodides are considered as promising reactants for atomic substitution to synthesize TMCs at low temperatures [34, 35]. It also shows a great advantage in growing non-layered TMCs (such as CdS) with an ultrathin thickness. For instance, using layered CdI_2_ nanosheets as host materials, Zhao et al. realized the synthesis of non-layered CdS nanosheets in a S rich vapor at 280–300 °C (Fig. 20a–d) [35]. The obtained CdS nanosheets possess a submillimeter size and atomic layer thickness of 2 nm, which was the smallest thickness as reported (marked in square region in Fig. 20c). Combining with DFT calculations, the formation process of CdS was proposed as follows: (i) replacement of I atoms by S atoms, (ii) formation of Cd–S chemical bonds, and (iii) lattice compression along the thickness-direction of CdS. In 2023, Zhang epitaxially grew metal iodide on wafer-scale substrates at a temperature below 400 °C, which was much lower than the direct growth of TMCs [34]. This metal iodide was then converted into TMCs by overcoming a small energy barrier of substitution between iodine and other chalcogens, as shown in Fig. 20e. By this two-step method, 17 different high-quality wafer scale TMCs (*M* = In, Cd, Cu, Co, Fe, Pb, Sn and Bi) were successfully synthesized, including metal sulfides, metal selenides, metal tellurides and metal chalcogenide alloys. Since most metal iodides are layered structures, during the transition to non-layered TMC crystals, the van der Waals gap between the iodide layers disappears, resulting in a reduction in the thickness of TMCs. As shown in Fig. 20f, the thickness of the obtained CdS sheet was almost half of that of the original CdI_2_ sheet. This method provides a new solution for the fabrication of ultrathin non-layered TMC materials with a large scale. Additionally, the substitutions involving transition metal iodide introduces a novel strategy for the synthesis of 2D TMCs synthesis at temperature below 400 °C.

#### Nitrogen-Chalcogen Substitution

**Fig. 21:**
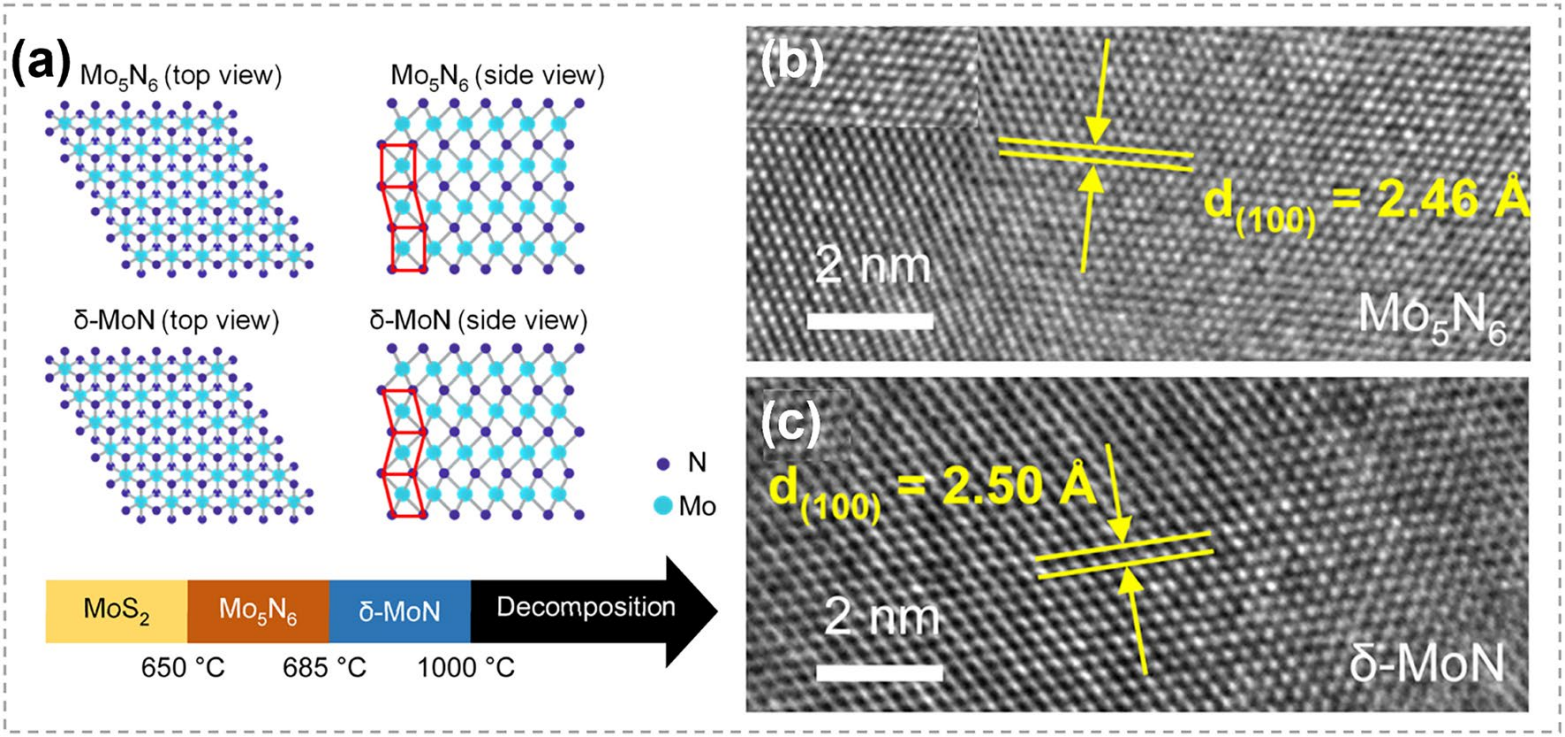
Synthesis of nitrides by atomic substitution. **a** Crystal structures of Mo_5_N_6_ and δ-MoN. **b, c** HRTEM images of the obtained Mo_5_N_6_ and δ-MoN. Reproduced with permission [165]. Copyright 2022, American Chemical Society

Through atomic substitution, layered 2D TMCs can also be converted into non-layered materials with an ultra-thin thickness. As illustrated in Fig. 21a, by using layered MoS_2_ as reactants, Ling et al*.* synthesized two phases of molybdenum nitride (Mo_5_N_6_ and δ-MoN) at different temperatures [165]. The morphology and 2D feature of host materials were inherited after the substitution of S with N, resulting in non-layered molybdenum nitrides. HRTEM image in Fig. 21b, c shows that the obtained Mo_5_N_6_ and δ-MoN were highly crystalline with a thickness of only a few nanometers. These obtained ultra-thin nitrides showed ohmic contacts with Cr/Au electrodes, indicating their promising applications in nanoelectronics.

### Synthesis of 2D TMC Heterostructures

As one of the most important structures in semiconductor industry, heterostructures are essential in high-speed electronic and optoelectronic devices [166–169]. The majority of TMC heterostructures are composed of a series of materials in which either the transition metals or chalcogens are different. This allows for the exploration of different material combinations and properties [170–172]. Traditional preparation techniques for heterostructure refer to stitching or stacking different TMC components together via a direct growth procedure, such as CVD heteroepitaxial synthesis [173–175]. Generally, the designability of components and structures are restricted. In contrast, atomic substitution offers a powerful tool for precisely tailoring the interfaces and optimizing the performance of TMC heterostructures.

**Fig. 22:**
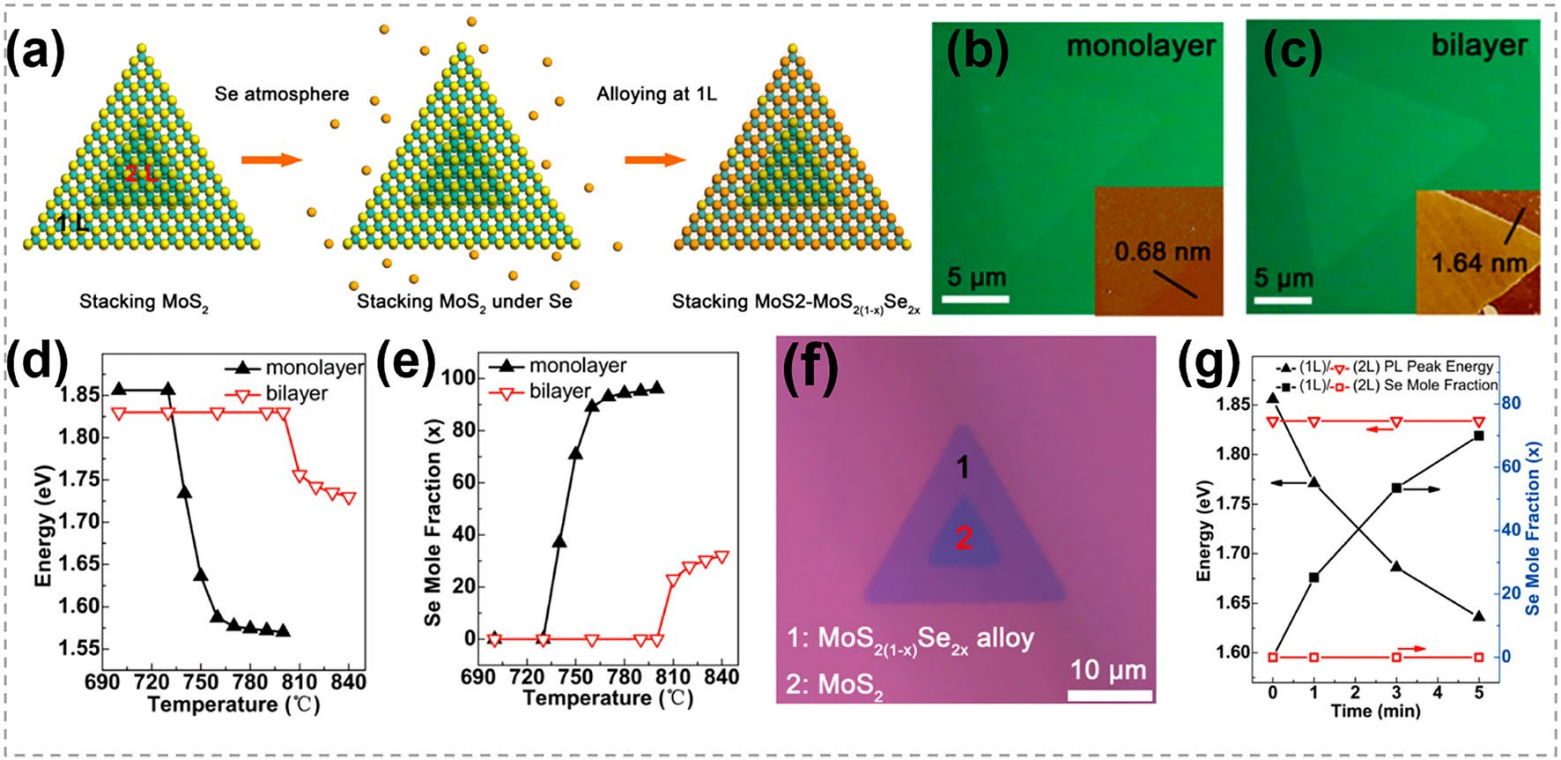
Heterostructure synthesized by chalcogenides substitution. **a** Schematic diagram of the preparation of MoS_2_-MoS_2(1-x)_Se_2x_ heterostructure by the Se substitution in a designed stacking MoS_2_ nanosheet. **b, c** Optical images of monolayer and bilayer MoS_2_ nanosheets after substitution with the corresponding AFM images shown in inset. **d, e** Bandgap values and compositions of the two sheets as a function of annealing temperature. **f, g** Optical image, bandgap values and compositions of the obtained MoS_2_-MoS_2(1-x)_Se_2x_ heterostructures. Reproduced with permission [23]. Copyright 2017, American Chemical Society

By performing layer-selected atomic substitution of MoS_2_ bilayer, Li et al. realized the synthesis of MoS_2_-MoS_2(1-x)_Se_2x_ heterostructures [23]. Both the preparation of MoS_2_ bilayer and substitution process were performed by CVD, as shown in Fig. 22a–c. The substitution temperature for monolayer was 740 °C, while that for the bilayer was 810 °C (Fig. 22d–e). This makes it possible to control the substitution process by modulating the reaction temperature. Figure 22f shows that the achieved MoS_2_-MoS_2(1-x)_Se_2x_ heterostructures were highly crystalline with a sharp interface. The photoluminescence energy and the corresponding Se composition of monolayer and bilayer regions at 750 °C as a function of annealing time is given in Fig. 22g. It was demonstrated that S atoms in the monolayer region underwent gradual substitution by Se atoms, whereas the bilayer region remained highly stable. Therefore, effective control over the temperature and duration of the substitution process allows for well-defined morphology of the synthesized heterostructure. These composition-tannable heterostructures hold great potentials in 2D fundamental physical research as well as the development of functional electronic and optoelectronic devices.

**Fig. 23:**
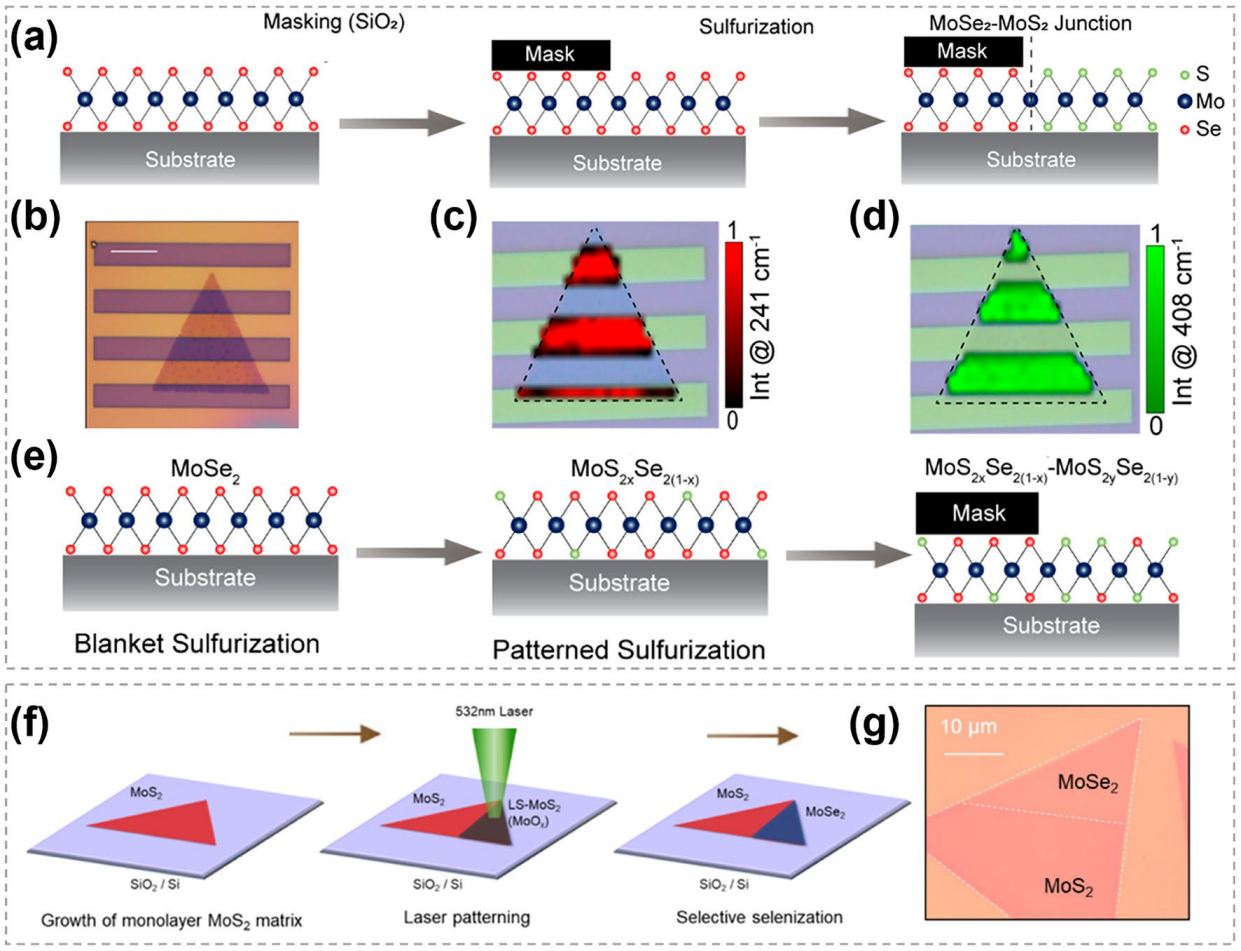
The selective substitution of TMC heterostructure synthesis. **a** Schematic showing the heterostructures synthesis protocol based on the sulfurization of patterned MoSe_2_ monolayers. **b** Optical image of a representative lateral heterostructure. **c, d** Mapping intensity of the A_1g_ Raman modes of MoSe_2_ in (**b**) at 241 cm^–1^ and MoS_2_ at 408 cm^–1^, respectively. **e** Schematic showing the realization of an (x, y)-heterostructure with x < y. Reproduced with permission [176]. Copyright 2020, American Chemical Society. **f** Schematic illustrations showing the formation process of MoS_2_-MoSe_2_ by laser patterning and selective selenization. **g** Microscopy images after the process illustrated in **f**. Reproduced with permission [177]. Copyright 2021, American Chemical Society

Patterned growth is a powerful method for customizing the morphology of heterostructures, which involves patterning the base material with a desired shape and a subsequent selective substitution. Figure 23a depicts the lithography process for the synthesis of lateral TMC heterostructures [176]. To realize selective substitution, a part of the MoSe_2_ film was protected by SiO_2_ mask. A MoSe_2_-MoS_2_ junction was achieved after well annealing in an S atmosphere. Figure 23b illustrates the optical image of the synthesized heterostructure, and Fig. 23c, d shows the Raman mapping intensity. In comparison, a uniform MoS_2x_Se_2(1-x)_ alloy was obtained without protection by mask, and the substitution ratio ‘‘x’’ in MoS_2x_Se_2(1-x)_ alloy could be tuned through controlling the extent of sulfurization. Based on this, alloyed heterostructures could be further synthesized by selectively sulfurizing MoS_2x_Se_2(1-x)_ to MoS_2x_Se_2(1-x)_-MoS_2y_Se_2(1-y)_ heterostructure, as illustrated in Fig. 23e. Similarly, Mahjouri-Samani et al*.* introduced an electron beam lithography method to selectively replace Se atoms in a mask-patterned MoSe_2_ flakes, and MoSe_2_-MoS_2_ heterojunction array was obtained under pulsed laser vaporization of sulfur [24]. Such lithography technology offers flexibility and benefits for both the precise device design and subsequent performance regulation. In 2021, Wang et al. realized the selective substitution by laser-induced oxidation without the assistance of masks [177]. As shown in Fig. 23f, monolayer MoS_2_ was synthesized via CVD and then scanned by a laser. The laser-scanned MoS_2_ could be oxidized into MoO_x_ and then selenized in an Ar/H_2_ atmosphere (Fig. 23g). During selenization, the unoxidized region remained intact, because MoO_x_ is easier to be selenized at a lower temperature as compared with MoS_2_.

**Fig. 24:**
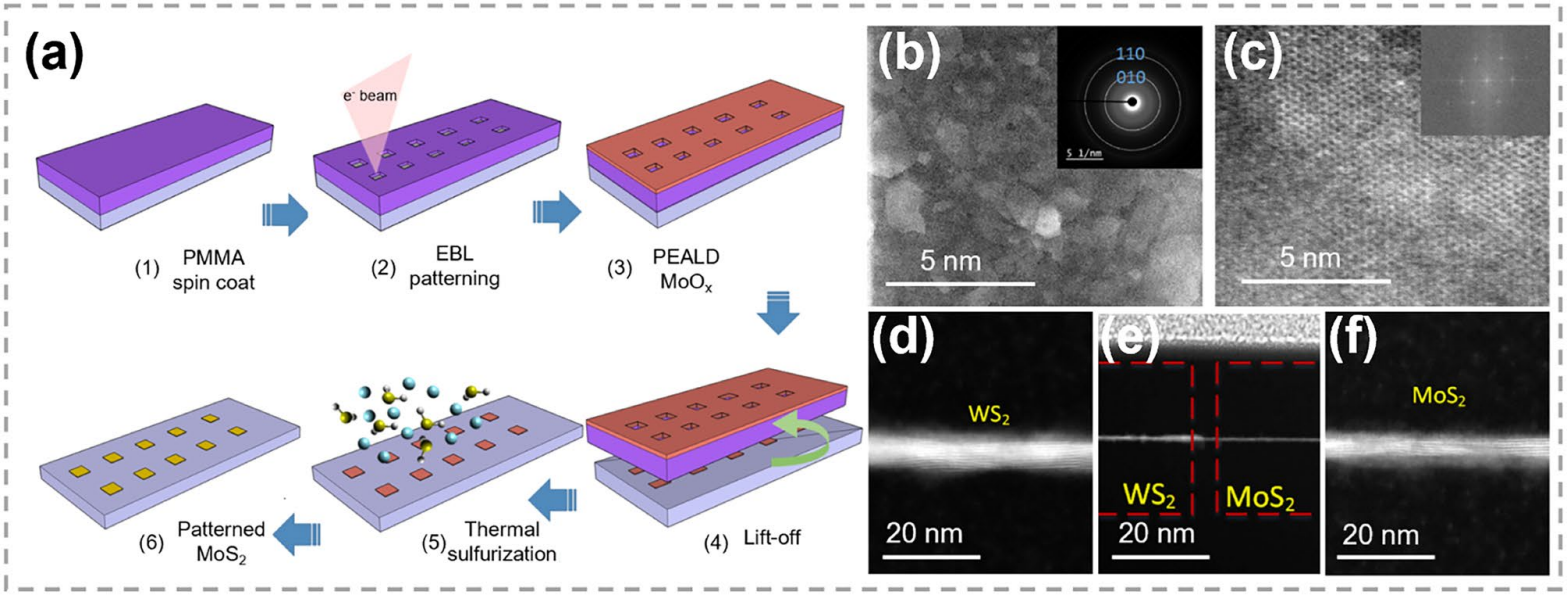
Patterned synthesis of TMC by substituting oxygen atoms with chalcogens. **a** Scheme of the atomic layer deposition enabled growth of large area patterned MoS_2_. **b** STEM image showing the uniform coverage of the polycrystalline MoS_2_ film. The inset shows a selective area electron diffraction pattern from the same sample which confirms the polycrystalline. **c** HR-STEM image of MoS_2_. **d-f** Low magnification cross-sectional HR-STEM characterization of **e** WS_2_/MoS_2_ junction and magnified cross-sectional HR-STEM images of **d** WS_2_ and **f** MoS_2_ regions. Reproduced with permission [178]. Copyright 2020, IOP Publishing

Metal oxide thin films were also used for the patterned synthesis of TMC heterostructures [178]. As illustrated in Fig. 24a, the MoO_x_ thin film was deposited on the patterned region of SiO_2_/Si substrates with plasma enhanced atomic deposition, the obtained metal oxide was then sulfurized by thermal treatment. STEM and high-resolution scanning TEM images in Fig. 24b, c confirmed the realization of polycrystalline MoS_2_ films. Importantly, by using a designed MoO_x_/WO_x_ heterostructure as the reactant, a lateral MoS_2_/WS_2_ heterostructures was obtained after sulfurization (Fig. 24d–f). This research provides an effective pathway for the synthesis of large-area 2D M_a_X_2_-M_b_X_2_ (M_a_ and M_b_ represent different metals) heterostructures.

### Synthesis of 2D TMC Janus Structures

**Fig. 25:**
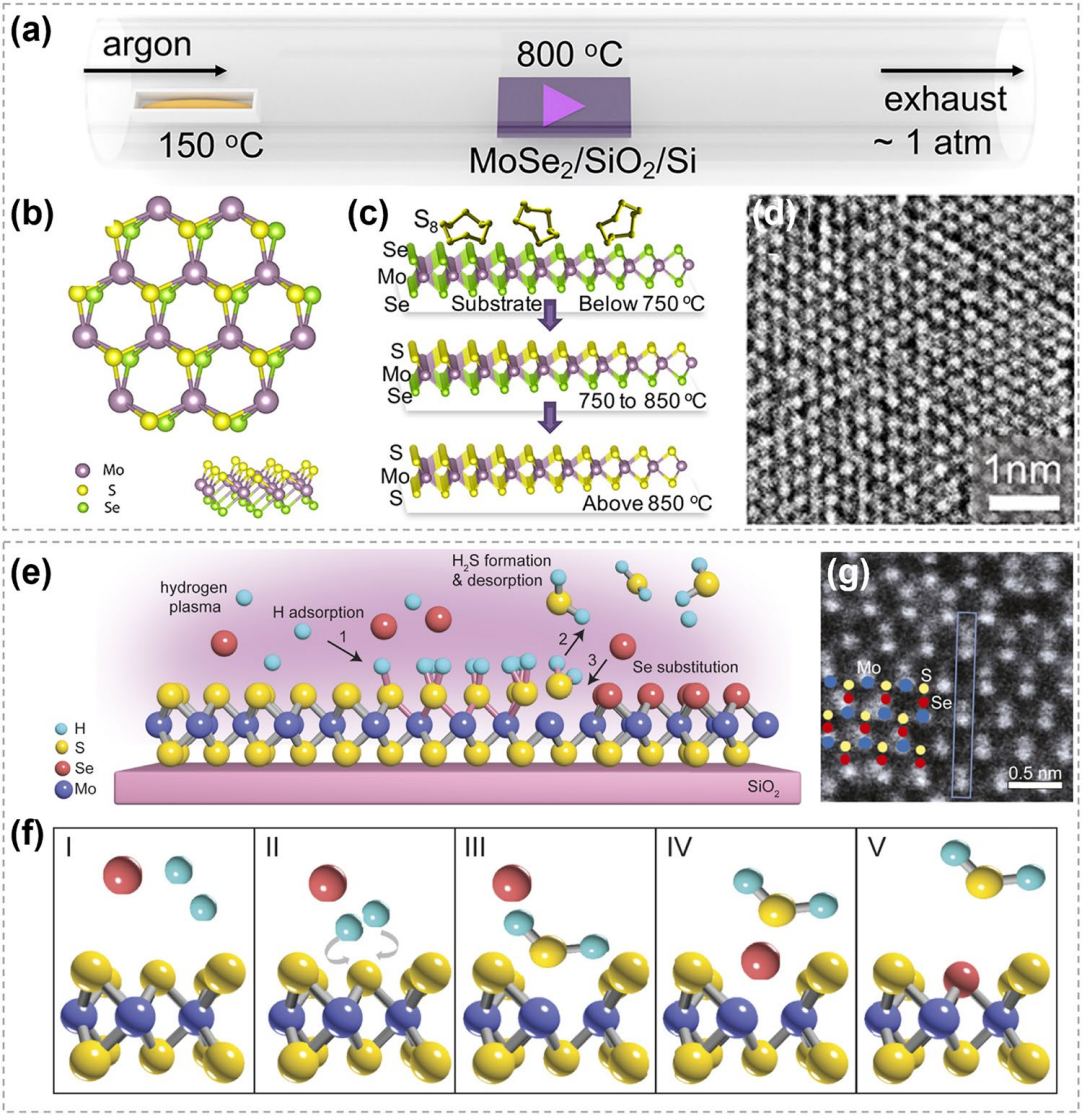
Synthesis of 2D TMC Janus structures by atomic substitution. Synthesis of 2D TMC Janus structures by atomic substitution. **a** Schematic illustration of the reaction setup for Janus SMoSe monolayer. **b** Different views of an eight-unit-cell Janus SMoSe monolayer. **c** Proposed mechanism for the sulfurization of monolayer MoSe_2_ at different temperatures. **d** HRTEM image of the Janus SMoSe lattice. Reproduced with permission [182]. Copyright 2017, American Chemical Society. **e** Schematic showing the asymmetric MoSSe monolayer structure. **f** Schematics showing key reaction steps for room-temperature atomic-layer substitution process. **g** Tilted ADF-STEM image of the Janus MoSSe sample. Reproduced with permission [156]. Copyright 2021, NATL ACAD SCIENCES

Janus structure stands out as a unique form of heterostructures, which is characterized by its asymmetric atomic arrangement [179]. The distinctive asymmetry gives rise to intriguing properties, including strong Rashba spin splitting, heightened second harmonic generation response, enhanced piezoelectric polarizations, as well as improved catalytic activity [180, 181]. Zhang et al*.* reported the synthesis of monolayer Janus SMoSe by selectively substituting Se atoms in the top layer of MoSe_2_ with S, as illustrated in Fig. 25a, b [182]. The S source, generated by heating sulfur powder to 150 °C, was introduced to react with MoSe_2_. The top Se layer of MoSe_2_ was exposed to the atmosphere and in direct contact with S atoms. As a result, Se atoms in the top Se layer underwent a rapid substitution by S atoms, while the bottom Se layer was maintained. It is worth noting that the substitution process was highly dependent on the experimental temperature (Fig. 25c). Within the temperature range of 750–850 °C, a Janus SMoSe monolayer with a 2H lattice structure was achieved (Fig. 25d). However, when the temperature exceeded 850 °C, the substitution extended to the bottom Se layer, which led to the formation of MoS_2_.

In 2021, Guo et al. proposed a room-temperature atomic-layer substitution method and synthesized Janus TMC structures successfully [156]. As illustrated in Fig. 25e, hydrogen plasma was applied to remove S atoms from the top S layer of a pre-grown MoS_2_, and Se vapor was supplied simultaneously to occupy the S vacancies. The detailed substitution process is schematically shown in Fig. 25f: (i) the initial state of MoS_2_, (ii) the adsorption and diffusion of two H atoms near a S atom, (iii) the formation of H_2_S, (iv) the desorption of H_2_S, and (v) the occupation of the vacancy site by Se atom. Annular dark-field STEM image in Fig. 25g confirmed the formation of the Janus structure, where Se atoms occupied one side of the monolayer SeMoS and S atoms occupied the other side.

Both of the above researches have realized the synthesis of Janus, though different methods adopted. The precious control of removing single-layer chalcogens is identified to be the crucial factor in both experiments. Compared with one-step synthesis method, post-treatment approach offers a better control over the morphology of the products. The synthesis of Janus structure opens up a new paradigm for the delicate design of artificial 2D materials, potentially unveiling unprecedented electronic, photonic and mechanical properties that are unexplored in nature.

### Synthesis of 2D TMC Alloys

**Fig. 26:**
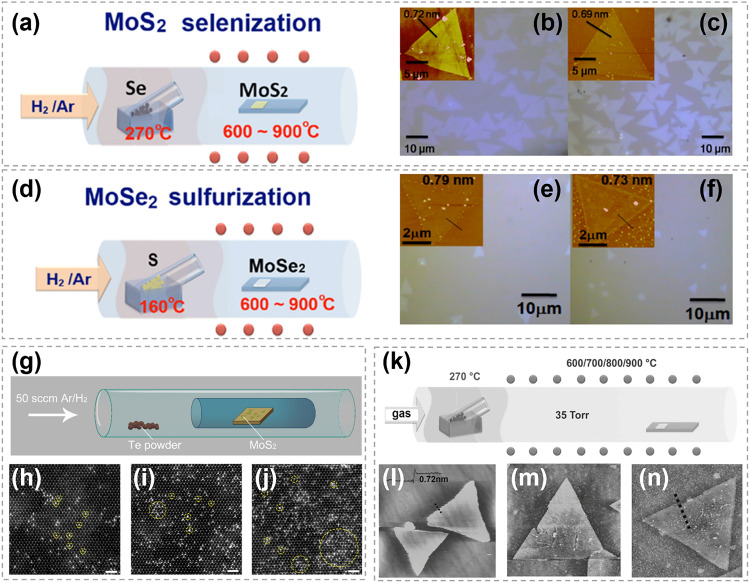
Synthesis of TMC alloys by atomic substitution. **a, d** Schematic illustration of the experimental set-up for the selenization/sulfurization process. **b, e** Optical micrographs for the as-synthesized MoS_2_/MoSe_2_. **c, f** Selenized (sulfurized) MoS_2_/MoSe_2_ (at 800 °C) on sapphire substrates. Reproduced with permission [150]. Copyright 2014, Frontiers Media S.A. **g** Schematic setup for the post-growth substituting. **h-j** Atomically resolved ADF-STEM images showing the atomic structure of Te-doped MoS_2_ monolayer at different growth temperature. A number of bright sites corresponding to the local Te sites can be distinguished (marked with yellow circles). Scale bar: 1 nm. Reproduced with permission [151]. Copyright 2018, IOP Publishing. **k** Schematic illustration for the selenization process. **l-n** AFM images for the MoS_2_ flake **l** before and **m** after**, n** selenization at 800 °C. Reproduced with permission [184]. Copyright 2014, John Wiley and Sons

Without patterning or delicate control over experimental conditions, the partial substitution treatment of 2D TMC materials commonly results in 2D TMC alloys [183]. Li et al. have explored two strategies for the synthesis of monolayer MoS_2x_Se_2(1-x)_ alloys by CVD methods [150], including the selenization of MoS_2_ and the sulfurization of MoSe_2_ (Fig. 26a, d). Optical images for MoS_2_ flakes before and after selenization are shown in Fig. 26b, c. It was found that S atoms were randomly and homogeneously substituted by Se, leading to the formation of a uniformly distributed MoS_2x_Se_2(1-x)_ alloy. Optical images for MoSe_2_ flakes before and after sulfurization are shown in Fig. 26e, f. Different from the substitution of S by Se, the substitution of Se by S preferred to occur along specific crystalline orientations of MoSe_2_, leading to the synthesis of MoSe_2_/MoS_2_ bi-phases. The optical band gaps of obtained MoS_2x_Se_2(1-x)_ alloys through the two different strategies were significantly different. Despite the selenization of transition metal disulfides presented a better control over the compositions and optical characteristics of monolayer TMC alloys, the different mechanisms of selenization and sulfurization are not clear.

Additionally, the tellurization of MoS_2_ was also explored (Fig. 26g). At a relatively low Te concentration, Te replaced S randomly, resulting in the formation of a monolayer MoS_2(1-x)_Te_2x_ alloy [151]. As the Te concentration increased to ~ 27.6%, local regions of Te enrichment accompanied with structure distortion were observed (Fig. 26h–j). Statistical analysis showed that Te substitution preferentially occurred in the top S sublattice of MoS_2_. Besides, hydrogen carrier gas played an indispensable role in the substitution process. In this research, the MoS_2(1-x)_Te_2x_ alloys with composition controllable were realized by regulating the amount of the reactants. Similarly, Li et al*.* reported the synthesis of MoS_x_Se_y_ flakes through selenizing MoS_2_ monolayers (Fig. 26k) [184]. As the temperature increased, there was a gradual substitution of S atoms with Se. The atomic force microscope (AFM) images of MoS_2_ flakes before and after selenization are shown in Fig. 26l–n, which proved the intact crystal structure after selenization. The band gaps of the resulting MoS_x_Se_y_ alloys were effectively tuned from 1.86 to 1.57 eV, depending on the Se content. This study provides a brief and straightforward method for engineering the band gap of 2D TMCs. Nevertheless, to enhance control over the composition of TMC alloys, there is a substantial need for a comprehensive understanding on the relative mechanisms of the substitution processes among different chalcogens.

**Fig. 27:**
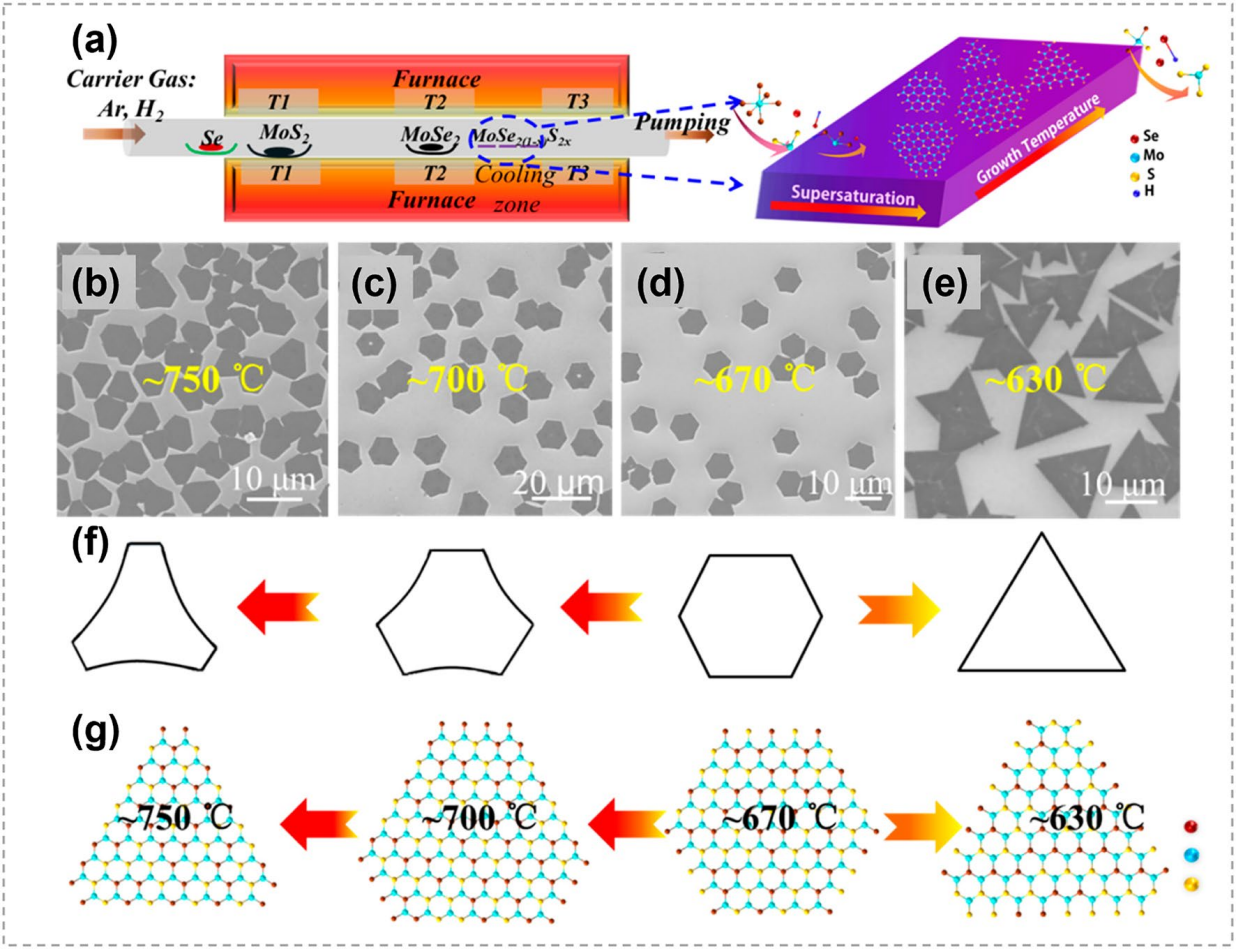
Controllable synthesis of MoS_2(1-x)_Se_2x_ alloys by atomic substitution. **a** Illustration of three-zone furnace setup for MoS_2(1-x)_Se_2x_ (*x* = 0.41–1.00) monolayer growth. **b**-**e** SEM images of MoS_0.78_Se_1.22_ domains obtained at different deposition temperatures. **f** Schematic illustration of different morphologies at different temperatures. **g** Atomic structures showing domains obtained at different temperatures. Reproduced with permission [185]. Copyright 2015, American Chemical Society

In addition to composition control, there is a strong demand for TMC materials with controllable morphology and domain size to further advance their applications. In Fig. 27, by tuning the temperature in physical vapor deposition (PVD) method, Feng et al*.* realized the synthesis of MoS_2(1-x)_Se_2x_ (*x* = 0.41–1.00) monolayer alloys with controlled edge orientation (Mo-zigzag and S/Se-zigzag edge orientations) and domain size [185]. By maintaining lower temperature gradients in the deposition zone, MoS_2(1-x)_Se_2x_ monolayer domains with size up to 20 μm were successfully obtained. This research provides valuable insights into the PVD growth of 2D TMC alloys, enabling control over composition, edge orientations as well as domain sizes.

It is worth noting that, by the atomic substitution methods, the obtained 0D and 1D MX alloys primarily belong to intermetallic alloys, while the achieved 2D MX_2_ alloys are classified as chalcogenide alloys. This may be attributed to the unique sandwich structure of MX_2_, where metal atoms are situated between two chalcogen layers, rendering the substitution between metal atoms more challenging.

### Synthesis of 2D Doped TMC Materials

Compared to alloys, doping involves the addition of guest atoms with much lower concentrations. Generally, doping is focused on introducing specific properties or characteristics to the host material without significantly altering its structure. Transition metal doping is highly demanded for enriching the properties and applications of TMC materials [183, 186].

**Fig. 28:**
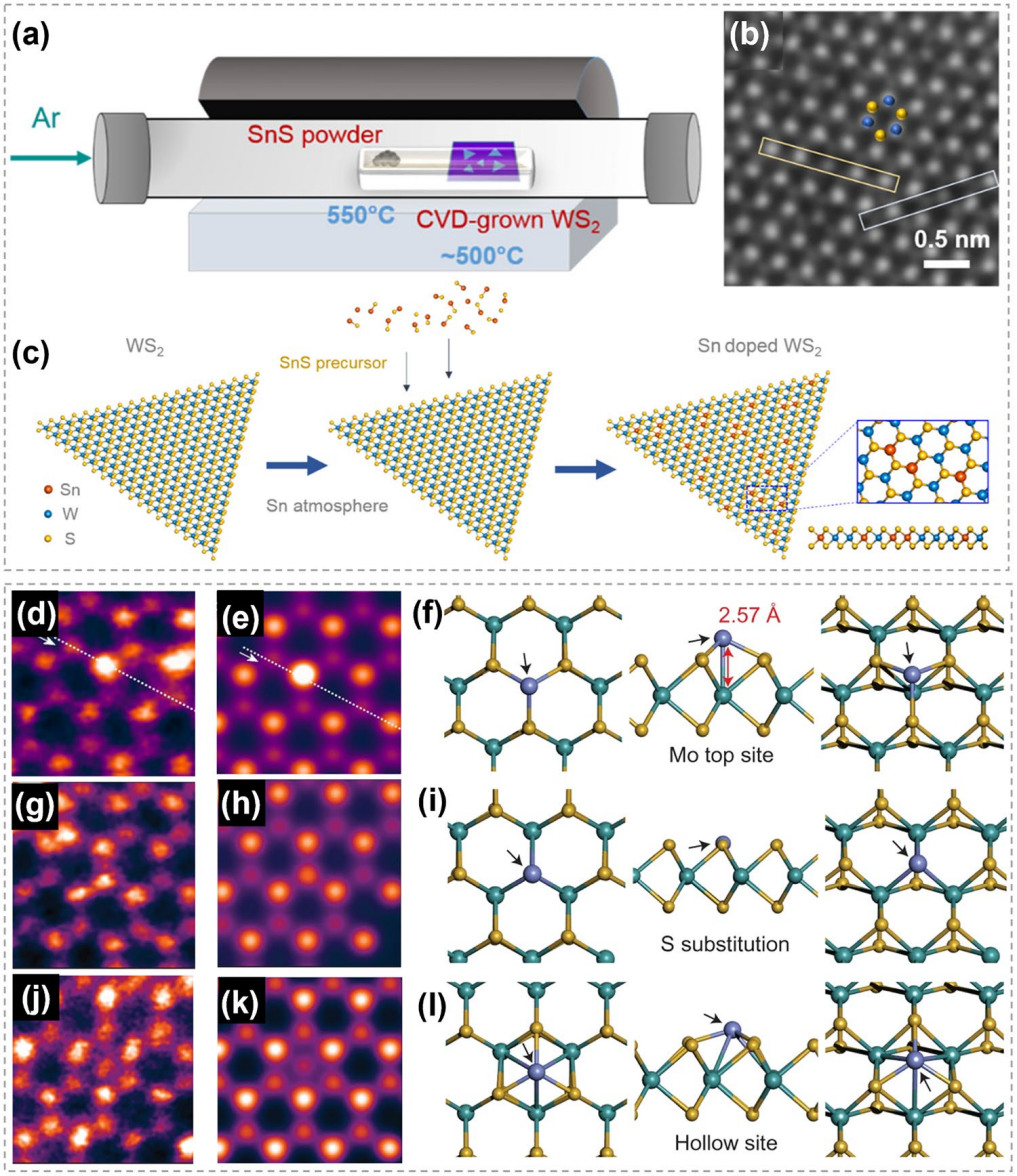
Metal atoms substitution doping of TMCs. **a** Schematic showing the thermal doping of Sn in WS_2_. **b** ADF-STEM images showing the area of Sn-doped WS_2_ monolayer. **c** Schematics showing the formation of Sn-doped WS_2_ monolayers. Reproduced with permission [155]. Copyright 2019, American Chemical Society. **d-f** ADF-STEM image, image simulation and atomic model from density functional theory (DFT) geometry optimization of the Co on Mo atop site. **g**-**i** HAADF image, image simulation from DFT geometry optimization and atomic model from geometry optimized DFT of a Co-substituted S site. **j**-**l** HAADF image, image simulation and atomic model from DFT geometry optimization showing Co in the hollow site. Reproduced with permission [154]. Copyright 2017, Springer Nature

Recently, Chang et al. developed a two-step process for the doping of WS_2_ monolayer by Sn (Fig. 28a–c) [155]. Firstly, 2D WS_2_ monolayers were synthesized by conventional CVD. Secondly, a post-thermal doping process was conducted by annealing WS_2_ monolayers in a Sn-rich environment, which is produced by heating SnS precursors. HRTEM image in Fig. 28b confirmed that the high crystalline of WS_2_ monolayer was maintained after a small proportion of W atoms were substituted by Sn. Electrical measurements indicated that the WS_2_ after Sn doping showed n-type doping behaviors.

Through a hydrothermal treatment of single-layer MoS_2_ by Co(thiourea)_4_^2+^ complex, Tsang and co-workers realized isolated Co atom doping in MoS_2_ [154]. The loading of Co in monolayer MoS_2_ was 1.8 wt%. As shown in Fig. 28d–l, isolated Co atoms incorporated into MoS_2_ basal plane showed three configurations: Co locating on atop site of Mo, Co substituting S site, and Co locating in the hollow site. Simulation results showed that Co atoms located on atop site of Mo atoms were the most energetically favorable. In another case recently reported by this group, the doping content of Co was raised to 3.0 wt% by increasing the Co/Mo ratio in the mixed solution in hydrothermal method [187]. This method can be applied to various transition metals, such as Fe, Co, Ni, Ru, Pd, and Ag, as mentioned in their study. It is noted that Dolui et al. employed ab initio calculations to explore various dopants at substitution and adsorption sites in monolayer MoS_2_ [183]. Nb, Zr, and Y atoms were found to act as p-type dopants and prefer to substitute the Mo lattice sites, since they had a lower number of valence electrons than Mo. These researches provide new strategies for the structural modification of 2D TMCs by atom doping and lays a foundation for the rational design of 2D TMCs to satisfy various requirements.

**Fig. 29:**
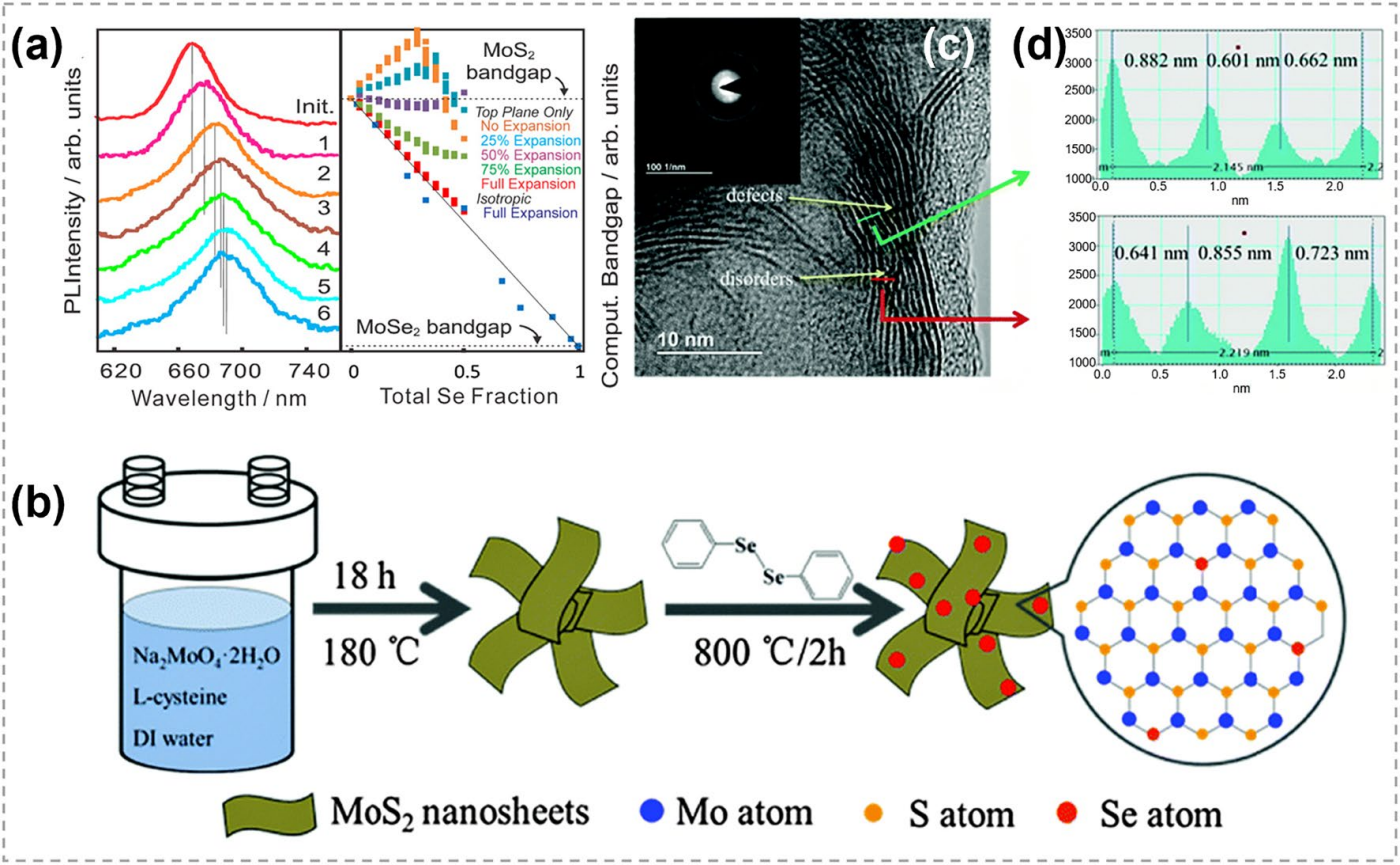
Chalcogens substitution doping in TMCs. **a** Normalized room-temperature photoluminescence spectra of a single-layer MoS_2_ film after sputtering and diselenodiphenyl insertion cycles. Reproduced with permission [188]. Copyright 2014, American Chemical Society. **b** Schematic illustration of the synthesis procedure for the Se-doped MoS_2_ nanosheets. **c** HRTEM image and (inset) the corresponding selected area electron diffraction patterns of Se-doped MoS_2_ nanosheets. **d** The lattice distances of the labeled green and red lines in **c**. Reproduced with permission [189]. Copyright 2015, The Royal Society of Chemistry. (Color figure online)

Chalcogen doping has also been extensively explored. Ma et al. employed a conventional CVD technique to grow monolayer MoS_2_, followed by the selective removal of a certain proportion of sulfur atoms by Ar plasma [188]. Diselenodiphenyl was then used to perform Se doping in MoS_2_ crystals. The photoluminescence spectra in Fig. 29a showed evidence for the successful doping of Se atoms, and the composition-based band gap modulation was consistent with theoretical predictions. Combining this method with patterning, it is possible to realize doping at desired locations. Se-doped MoS_2_ nanosheets could also be synthesized by annealing MoS_2_ with diphenyl diselenide at 800 °C (Fig. 29b) [189, 190]. The Se contents after substitution was approximately 6%. It's worth noting that there was an expansion of the interlayer spacing from 0.6 to 0.88 nm after such a small Se doping, implying an increase in the degree of disorder (Fig. 29c, d), which resulted in an increase in the number of active edge sites and therefore an improved catalytic activity [189]. These research results show that introducing doping to modulate chemical composition provides routes to improve the electronic, optical and chemical properties of TMCs.

**Fig. 30:**
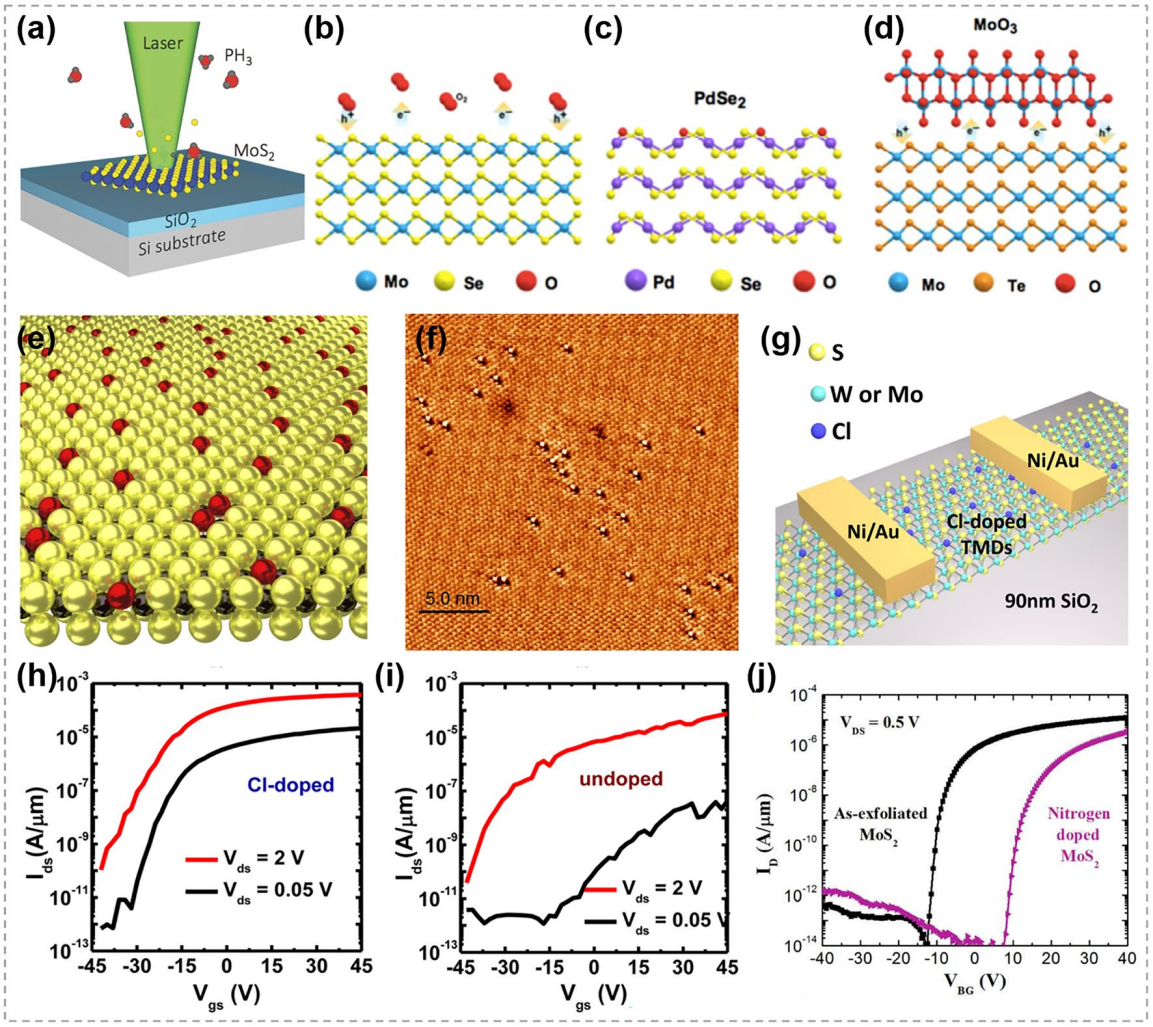
Nonmetal elements substitution doping in TMCs. **a** Schematic diagram of the laser-assisted doping method. Reproduced with permission [197]. Copyright 2016, John Wiley and Sons. **b** Illustration showing charge transfer from oxygen molecules. **c** Isoelectronic substitution of chalcogen atoms in TMCs. **d** Illustration showing charge transfer from oxide. Reproduced with permission [198]. Copyright 2020, Springer Nature. **e** Schematic representation of atmospheric oxygen atoms getting adsorbed on a MoS_2_ monolayer. **f** Scanning tunneling microscope image of O atoms (bright spots) absorbed at S vacancies (dark triangles) sites. Reproduced with permission [199]. Copyright 2018, Springer Nature. **g** Schematic of Cl-doped few-layer WS_2_ back-gate field-effect transistors (FETs). **h** Transfer characteristics of the device (Cl-doped). The *I*_on_/*I*_off_ ratio is about 4 × 10^6^ and 3 × 10^7^ at *V*_ds_ of 2 and 0.05 V, respectively. **i** Transfer characteristics of the device (undoped). The *I*_on_/*I*_off_ ratio is about 2 × 10^6^ and 1.1 × 10^4^ at *V*_ds_ of 2 and 0.05 V. Reproduced with permission [194]. Copyright 2014, American Chemical Society. **j** *I*_DS_-*V*_GS_ characteristics of multilayer nitrogen-doped MoS_2_ FET. Reproduced with permission [195]. Copyright 2016, American Chemical Society

In addition to metals and chalcogens, other elements have also been explored as dopants to regulate the electrical properties of TMCs, such as H [21, 191], O [192, 193], Cl [194], N [195], P [196], etc. In Fig. 30a, Kim et al. proposed a strategy of site-selective P doping in ultrathin MoS_2_ through laser-assisted reaction [197]. This method could realize the precise control of doping both temporally and spatially. In Fig. 30b–d, Liang et al. explored oxygen induced controllable p-type doping in a series of 2D TMCs such as MoTe_2_, WSe_2_, MoSe_2_, PtSe_2_ and PdSe_2_ [198]. Three possible mechanisms for promoting p-type doping in 2D TMCs were proposed: charge transfer from absorbed oxygen molecules, formation of surface oxide and substitution of sulfur atoms. Peto et al. investigated the process of oxygen substitution to S in MoS_2_ (Fig. 30e, f) [199]. O atoms were found to spontaneously and slowly bind with the basal plane to replace S atoms one by one. The oxygen substitution sites on MoS_2_ surface could act as the single atom reaction centers, greatly improving the catalytic activity for the electrochemical hydrogen evolution reaction. This study provides new ways for engineering 2D TMC electrocatalysts with single O-atom active sites. Yang et al. proposed a simple and convenient way to realize Cl-doping in mechanically exfoliated few-layer (3.5–5 nm) WS_2_ and MoS_2_ [194]. In this method, TMC flakes were soaked in undiluted 1,2-dichloroethane at room temperature for more than 12 h. Figure 30g shows the schematic of the structure of Cl-doped WS_2_ (MoS_2_) back gate field-effect transistors (FETs). The observed negative threshold voltage shift for WS_2_ FETs after 1,2-dichloroethane treatment proved the n-doping effect (Fig. 30h, i). It was also found that the contact resistance was reduced after doping, which was beneficial for improving the performance of 2D nano electron-device.

Doping TMCs by N and P elements has been achieved by using post-growth treatments including NH_3_ plasma treatment [200], N_2_ plasma treatment [195, 201], annealing in NH_3_ [202], and PH_3_ plasma treatment [196]. Azcatl et al. found that the threshold voltage of the transfer curve of MoS_2_ FETs shows a positive shift after N_2_ plasma treatment, indicating that N substitution doping induced the p-type doping effect (Fig. 30j) [195]. Nipane et al. also reported that p-type doping could be achieved in few-layer MoS_2_ by PH_3_/He plasma treatment [196].

In this section, the applications of atomic substitution strategy in 2D TMCs were reviewed. Different from the synthesis of 0D and 1D TMCs that occurs in solutions, atomic substitution approaches employed in 2D TMCs typically take place in a vapor atmosphere. To date, various 2D TMCs with desired compositions and morphologies, such as binary compounds, heterostructures, Janus, and alloys, have been realized by precious control on temperature, reaction time and chemical environment of the substitution process. It is worth noting that this strategy also makes it possible to synthesis 2D TMC structures at low temperatures by choosing appropriate reactants. However, the exploration in the controllable synthesis is most concentrated on the experiments. The understanding of substitution mechanisms between various atoms, particularly those involving different chalcogens, remains unclear. Hence, there is a need to provide a systematic theoretical analysis of the substitution process, grounded in the mechanisms underlying these various substitution categories.

## 3D Materials

**Fig. 31:**
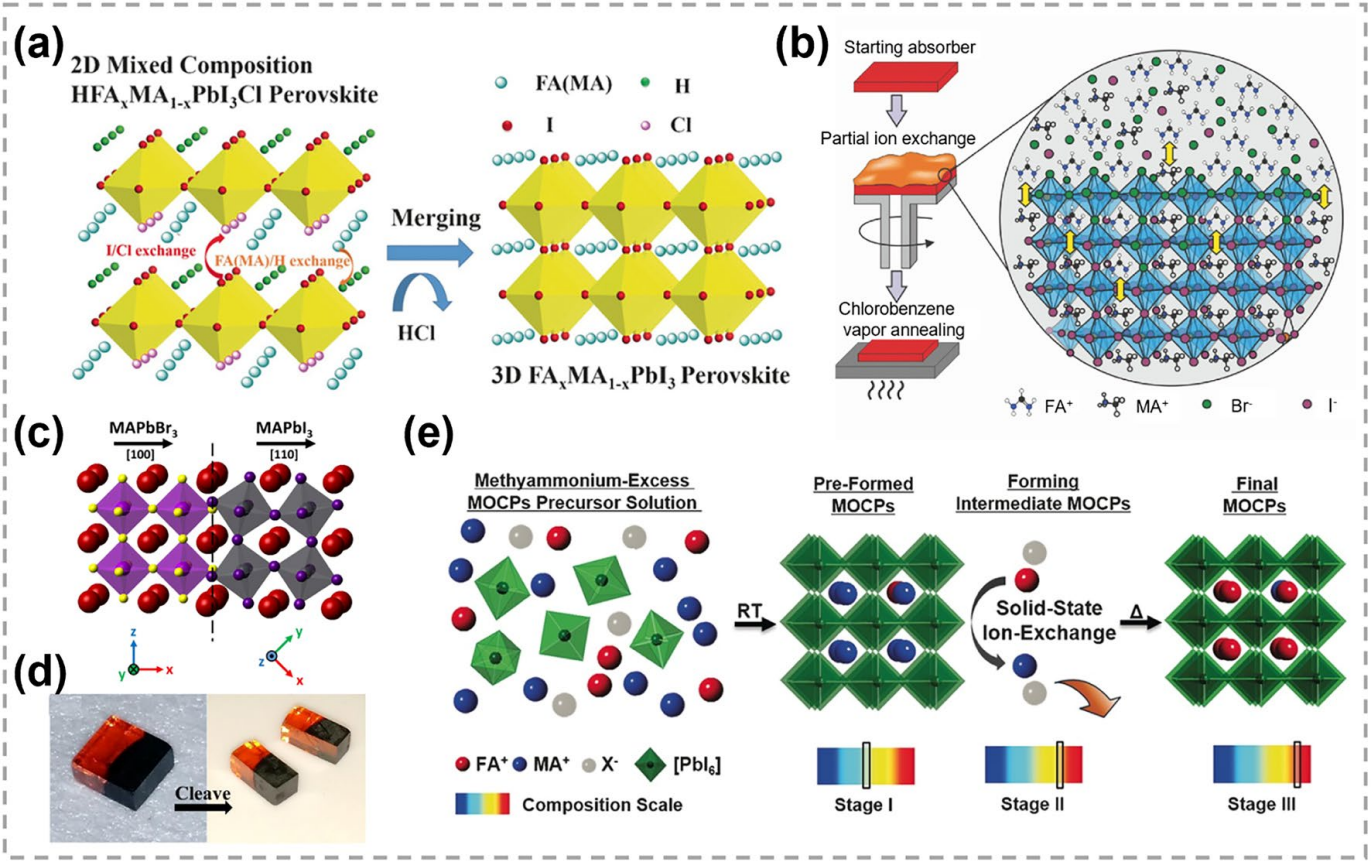
Atomic substitution in 3D materials. **a** Schematic diagram showing the transformation of 2D mixed HFA_x_MA_1-x_PbI_3_Cl into 3D FA_x_MA_1-x_PbI_3_ (MA = methylammonium, FA = formamidinium). Reproduced with permission [203]. Copyright 2016, John Wiley and Sons. **b** Illustration showing the preparation of compositionally graded mixed-cation lead mix-halide perovskite absorbers. Reproduced with permission [204]. Copyright 2018, John Wiley and Sons. **c, d** Atomic structure and optical images of MAPbBr_3_/MAPbI_3_ heterostructures. Reproduced with permission [205]. Copyright 2016, American Chemical Society. **e** MA-mediation synthesis of FA_1−x_MA_x_PbI_3_ mixed-organic-cation iodide perovskite from MA^+^-excess precursor solutions. X^−^ denotes an anion that does not conform with the structure. Reproduced with permission [206]. Copyright 2017, John Wiley and Sons

Additionally, the atomic substitution method also exhibits considerable potential in the synthesis of 3D materials. In Fig. 31a, Li et al. [203] prepared mixed HFA_x_MA_1-x_PbI_3_Cl (MA = methylammonium, FA = formamidinium) perovskite precursor films, using stoichiometric [HCl + (1 − x)MAI + x FAI + PbI_2_] solutions, and realized a structural conversion from 2 to 3D. The designed HFA_x_MA_1-x_PbI_3_Cl perovskite film, without long chain spacing, is inherently instable, which provides possibilities for H/FA(MA) and Cl/I ion exchange reactions. During the reactions, the exchanged H^+^ and Cl^−^ formed highly volatile HCl, readily releasing into the air. Meanwhile, the layer-structured 2D PbI_2_ precursor was transformed into a 3D structure, leading to the production of a phase-pure and high-quality 3D FA_x_MA_1-x_PbI_3_ perovskite. This research provides an effective and facile method for the synthesis of phase-pure halide perovskite with mixed-composition.

By the partial anion exchange method, Fu et al. [204] prepared compositionally graded perovskite absorbers successfully. As illustrated in Fig. 31b, MAPbI_3_ was prepared first as the starting absorber. It was then treated by spin coating MABr solution at room temperature to induce halide ion-exchange, and finally the MABr-treated film was thermally annealed under a chlorobenzene vapor atmosphere to promote the diffusion and redistribution of ions. Consequently, a MAPbI_3-x_Br_x_ perovskite absorber with a compositionally graded layer was formed, featuring a continuous decrease in Br concentration throughout the entire thickness. This achieved perovskite absorbers show an improve of the photovoltaic performance and operational stability in near-infrared-transparent perovskite solar cells. Except for the synthesis of compositional grading structures, the partial exchange method can also facilitate the fabrication of heterostructures. Shewmon et al. [205] achieved MAPbBr_3_/MAPbI_3_ heterojunctions by immersing MAPbBr_3_ single crystals into a MAPbI_3_ solution (Fig. 31c, d). The newly formed MAPbI_3_ layers were shown to be single crystalline, with the exchange of halide ions dominating the formation process. This work paves the way for the future research on optoelectronic devices using single-crystal materials.

Apart from anions substitution reactions, cations exchange can also occur in 3D materials. For instance, Li et al. [206] synthesized FA_1−x_MA_x_PbI_3_ mixed-organic-cation iodide perovskite (MOCP) thin films by adding excess MA^+^ cations into the precursor solution. As illustrated in Fig. 31e, FA^+^, MA^+^, X^−^ and PbI_2_ were random and disordered in the precursor solution, here X^−^ anion must be not incompatible with the MOCP crystalline structure in order to avoid influencing the composition of the final MOCP. At the room temperature, MOCPs with a relatively large fraction of MA^+^ cations were formed (Stage I). With the following thermal-annealing step, ion-exchange reactions occurred between pre-formed MOCPs and the surrounding FA^+^ (Stage II), due to FA^+^-rich hybrid organic–inorganic perovskites are thermodynamically more stable at increased temperatures. With the depletion of FA^+^ cations outside the MOCP phases, phase-pure, uniform and precisely composed MOCPs were formed (Stage III). During the process, the composition of the as-formed MOCPs is dynamically tuned with the FA^+^/MA^+^ ratio. In summary, the atomic substitution method provides a possible strategy for the synthesis of intricate 3D materials with diverse compositions and morphologies.

## Conclusion and Prospect

In recent years, the atomic substitution method has emerged as a prominent technique for the fabrication and manipulation for low-dimensional TMC materials due to its capacity for precise control over morphologies, compositions and structures. So far, extensive research efforts have been conducted to understand the underlying mechanisms of atomic substitution. It has been revealed that defects in the host materials play a crucial role in initiating the substitution reaction. Various experimental factors, including the chemical environment, temperature, and crystal structure, have been identified as significant factors in the process of atomic substitution. Additionally, the structures and morphologies of the resulting materials are greatly influenced by the substituting elements, substitution ratios, and substitution positions. Based on the understanding on the fundamental mechanisms of atomic substitute, a large variety of high-quality low-dimensional TMC materials have been successfully synthesized. Nevertheless, delicate control for atomic substitution, especially in the case of 2D TMCs, is still in its early stages.

To achieve the ultimate goal of realizing TMC materials with well-defined structures and compositions, more efforts are still required: (i) In situ characterizations are highly demanded for providing valuable insights into atomic substitution. By employing in-situ techniques such as STEM, X-ray photoelectron spectroscopy, Raman, Fourier transform Infrared spectroscopy, and X-ray absorption spectra, the dynamic transformation and reconstruction of active sites can be effectively visualized, allowing for a comprehensive understanding on the nature of a typical reaction [207]. (ii) The close collaboration between theoretical studies and experiments is encouraged. Theoretical studies are indispensable for understanding the synthesis mechanisms of materials [208, 209], and further providing guidelines for controllable synthesis. We believe that with the close collaboration between theoretical studies and experiments, theories of atomic substitution will finally be established and controllable synthesis of TMC materials will be realized. Besides, the diversity of types and combinations within TMC materials results in a vast number of members within this family. This intricate nature presents a challenge in fully exploring TMC materials by experiments, in this case, high-throughput computing research will be quite helpful.

Atomic substitution offers advantages not only in precisely engineering and customizing the desired properties of TMC materials but also in potentially overcoming the limitations of direct synthesis methods. However, it should be noted that although atomic substitution has been successfully achieved in laboratory, there is still a significant gap toward its practical implementation. With a comprehensive understanding of the mechanisms behind atomic substitution, it is anticipated that various synthesis procedures will be further optimized in the near future, and simultaneously, large-scale production will be achieved.
